# Assessing the effects of pandemic risk on cooperation and social norms using a before-after Covid-19 comparison in two long-term experiments

**DOI:** 10.1038/s41598-024-53427-z

**Published:** 2024-02-09

**Authors:** Eva Vriens, Aron Szekely, Francesca Lipari, Alberto Antonioni, Angel Sánchez, Luca Tummolini, Giulia Andrighetto

**Affiliations:** 1 Institute of Cognitive Sciences and Technologies, Italian National Research Council, Rome, Italy; 2 Institute for Futures Studies, Stockholm, Sweden; 3 Collegio Carlo Alberto, Turin, Italy; 4 Department of Economic Analysis and Quantitative Economics, Universidad Complutense de Madrid, Madrid, Spain; 5 Grupo Interdisciplinar de Sistemas Complejos (GISC), Departamento de Matemáticas, Universidad Carlos III de Madrid, Leganés, Spain; 6 Instituto de Biocomputación y Física de Sistemas Complejos (BIFI), Universidad de Zaragoza, Zaragoza, Spain

**Keywords:** Environmental social sciences, Natural hazards, Risk factors

## Abstract

How does threat from disease shape our cooperative actions and the social norms that guide such behaviour? To study these questions, we draw on a collective-risk social dilemma experiment that we ran before the emergence of the Covid-19 pandemic (Wave 1, 2018) and compare this to its exact replication, sampling from the same population, that we conducted during the first wave of the pandemic (Wave 2, 2020). Tightness-looseness theory predicts and evidence generally supports that both cooperation and accompanying social norms should increase, yet, we mostly did not find this. Contributions, the probability of reaching the threshold (cooperation), and the contents of the social norm (how much people should contribute) remained similar across the waves, although the strength of these social norms were slightly greater in Wave 2. We also study whether the results from Wave 1 that should not be affected by the pandemic—the relationship between social norms and cooperation and specific behavioural types—replicate in Wave 2 and find that these results generally hold. Overall, our work demonstrates that social norms are important drivers of cooperation, yet, communicable diseases, at least in the short term, have little or no effects on either.

## Introduction

The sudden emergence of the Covid-19 pandemic uprooted conventional ways of behaving in virtually every domain of social life. Especially during the first wave (March–December 2020), large public policy efforts were launched to promote changes in social practices that aimed to mitigate the pandemic risk. Consequently, novel hygiene practices, the avoidance of social gatherings, alternative greetings habits, and different ways to work, study, and travel emerged. In addition to these shifts in intended and pandemic-related behaviours, more domain general behaviours may have shifted. Indeed, one effect of Covid-19 may have been to shift our cooperative behaviours (the willingness of individuals to incur a net cost in order to benefit others), and the social norms that sustain them but it is unclear how. On the one hand, widespread media reports in the first weeks of the pandemic suggest that there was a breakdown of cooperation, with self-interested behaviour looming large, as exemplified by the panic buying of masks, toilet paper, groceries, which led to depletion of essential resources for others^1–3^. There is indeed some evidence that trust in fellow citizens or in institutions decreased during the pandemic^4–6^ and that people became less cooperative when periods of lockdown or social isolation last longer^7^. On the other hand, extensive evidence paints a less gloomy picture, typically finding that shared experience with Covid-19 or other environmental disasters at the societal level have led to increases in cooperation, trust, and prosociality^8–16^. In light of this mixed evidence, the first question that we ask here is: Did the outbreak of the Covid-19 pandemic make people more or less cooperative? Second, we ask, are changes (if any) in cooperative behaviour also accompanied by changes in social norms that promote cooperation? Tightness-looseness theory^17^ implies that social norms tend to adapt to threats and support cooperation.

We address both questions using data from an incentivised behavioural experiment conducted before the Covid-19 pandemic (Wave 1, 2018) in Spain^18^ and combine this with data from the same experiment that we re-ran in June 2020, sampling from the same population in Spain, during the first year of the Covid-19 pandemic (Wave 2). By holding constant incentives across the studies, our experiment is able to identify whether cooperation and social norms changed even when subjects face identical in-game conditions across the two waves. And, by implementing incentivised and repeated measures of social norms, we can accurately identify whether behavioural changes were accompanied by social norm changes.

Evidence relevant to our first question—whether Covid-19 increased cooperation or not—comes from three sources. First, and most closely related to our work, recent research studies how donating to charities (or NGOs) and prosocial behaviour towards others, including altruism, changed during the Covid-19 pandemic. Typically, these studies find evidence consistent with increased donating and prosociality during the pandemic^7–12^. For instance, Grimalda et al.^8^ find in two samples, one from Italy and another from the USA, that people who were personally exposed to Covid-19 donated a larger proportion of a monetary bonus, which they received during the study, to a charity involved in Covid-19 relief efforts than people who were not directly exposed to Covid-19. Gambetta and Morisi^19^ use a panel survey with a survey experiment to show that individuals that were infected with Covid-19 reported higher trust in strangers than those not infected. Still, other research finds that donations and prosocial behaviour are only little affected^20^ or entirely unaffected by the pandemic^21^ and some even report that donations decreased with longer exposure to the pandemic^22^. So, while there appears to be a general tendency of increased prosociality, the results are not equivocal. And, because of variations in study design, it is unclear whether the different results reveal true differences or are artefacts of the design choices. Most notably, the majority of existing studies^7–9,19,22^, especially those using a behavioural experiment, do not have a pre-Covid baseline comparison and rely only on data collected following the emergence of the pandemic. Consequently, the picture remains far from clear.

The two other sources of evidence concern research that studies people’s responses to natural disasters and war violence. Starting with natural disasters, one study finds that the experience of a tsunami increased trust 5 years after the event^13^. Another recruited recent evacuees of the 2005 hurricane Katrina disaster and studied how they behave in two cooperation games to find that cooperation remains substantial despite the difficult circumstances^14^. Other research, instead, uses participant observation, interviews, and systematic document gathering efforts to understand how people responded to the same disaster. They find that prosocial behaviour was by far the primary response to this event, despite widespread media reports of massive antisocial behaviour^15^. As to wars, in a comprehensive meta-analysis of the topic, Bauer et al.^16^ report that prosocial behaviour in experimental games is consistently higher among people who have been affected by war violence than those less affected. Their evidence comes from 16 papers that collected data from a wide range of countries including Sierra Leone, Tajikistan, Uganda, Georgia, Israel, and 35 countries in Europe, the Caucasus and Central Asia. Overall, this literature too suggests that disasters and prosociality are positively related. Yet, disease threats, by their very nature, may be fundamentally different to floods, tsunamis, or war violence. In contrast to these, getting physically close to and helping others during a pandemic is risky because people are the vector of virus transmission. So, here too the result is somewhat inconclusive.

Concerning our second research question—whether social norms of cooperation changed in response to Covid-19—we draw on the tightness-looseness theory of cultures^17,23^. This influential paradigm of social norm change posits that societies that have experienced more frequent and stronger ecological and social threats—e.g., frequent disease, warfare, and environmental catastrophes—throughout their histories have developed tighter cultures with stronger social norms and low tolerance of deviant behaviour to maintain order and survive crises. In contrast, low threat histories produce loose cultures with weak social norms and a high tolerance for norm-breaking^17^. Hence this theory implies that social norms supporting behaviour will be stronger after the emergence of the Covid-19 pandemic. While this is an established theory supported by rich empirical research, nearly all of the existing evidence comes from cross-sectional country-level or regional-level comparisons that may suffer from issues of confounding^17,23–26^ (but see^27–30^ for exceptions that look across time). Causal evidence that social norms and cooperation change in response to threats is scarce.

A recent exception to this is a 30-day online experiment that causally demonstrates that social norms strengthen in response to risk^18^. In this study, conducted in June and September 2018 (Wave 1), 286 subjects participated in a collective-risk social dilemma^31^. In this setting, which was designed to capture key features of climate change prevention, participants can spend some share of their endowment to prevent the possibility of a collective loss. If people within a group collectively spend enough of their endowment and reach a threshold level, they avert the collective loss with certainty. If they do not reach the threshold, there is a probability that the collective loss occurs and they lose all of their endowments. Additionally, subjects’ social norms—their empirical and normative expectations^32^ concerning cooperation within their group—were extensively measured in an incentivised way and tested for causality. By changing the probability of the collective loss, this experiment was able to study how threat affects cooperative behaviour and social norms of cooperation, and found that cooperation increased in response to a higher risk of the collective loss and social norms became stronger (i.e. more consistent, accurate, and specific).

Here we contribute to the aforementioned literature by replicating the 2018 experiment in the context of Covid-19 as a physical threat with 293 new subjects (sampled from the same Spanish population) during the early stage of the Covid-19 pandemic (June 2020, Wave 2). At this time, use of masks was mandatory and access to public spaces was limited in Spain, but there was no full lockdown. We compared the results of this long-term (30-day) incentivised behavioural experiment to the first experiment (Wave 1). This allows us to compare the post-Covid results with the pre-Covid baseline. The incentivised measures of social norms provide rich and behaviourally-grounded evidence for social norm change or stability.

## Experimental design

**Figure 1:**
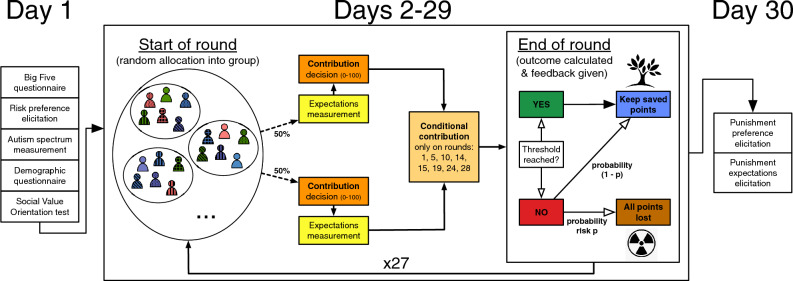
Experimental design. *Note:* Each day represents an experimental round. On day 1, participants performed four individual trait tests: Big Five, risk preference elicitation, Autism spectrum and Social Value Orientation, and completed a short questionnaire. From day 2 to day 29 participants were matched in groups of six people and interacted according to the collective risk social dilemma answering questions on their expectations (asked in a random order with 50/50 probability either before or after the contribution decision) and deciding their actual contribution and their conditional contributions. Conditional contributions were elicited on rounds 1, 5, 10, 14, 15, 19, 24, and 28. At the end of each round, participants kept their saved points if the threshold was reached, otherwise they lost all their round points with probability *p*. The outcome of the conditional contribution decisions was calculated based on the contributions and actual empirical and normative expectations of each group (see Supplementary Information Section ﻿S1 for details). Each day the groups were reshuffled. On day 30, participants went through a punishment phase in which their punishment behaviour and expectations were elicited. Subjects subsequently received information about their results and payment. Figure reproduced from^18^.

Both experiments follow an identical design (Fig. 1). Subjects participate in multiple stages of the experiment across 30 days. During day 1 they complete the Big Five questionnaire^33,34^, an incentivised risk preference elicitation task^35^, the autism spectrum quotient questionnaire^36,37^, the six item slider Social Value Orientation task^38^, and a demographic questionnaire. These measures serve to separate individual motives and preferences from social norm explanations. In the following 28 days (day 2–29), subjects are each endowed with 100 points, randomly allocated into groups of six each day, and are asked to contribute between 0–100 points to prevent a collective loss from occurring with a particular risk. If a total of 300 points were contributed (i.e., an average of 50 points per subject), the disaster was averted with certainty and subjects kept all points that they did not contribute. If the threshold of 300 points was not reached, with some probability subjects lost everything. After each round, subjects receive feedback about the contribution of all the other subjects in their group, whether the threshold has been reached or not, whether the catastrophe occurred or not, and their individual payoffs for that round. They were then randomly re-grouped and began a new round of the experiment. They were not informed about the behaviour of those outside their group. The experiment’s instructions are reported in section S1.1 in the Supplementary Materials.

Every day, we also measured subjects’ personal normative beliefs (“How much *one ought to* contribute to prevent the collective loss”), empirical expectations (“How much you think others in your group *actually contribute* to preventing the collective loss”), and normative expectations (“How much you think *others in your group think that one ought to* contribute to prevent the collective loss”)^32,39^. The latter two expectations are key elements of social norms whilst we measure the former in order to separate one’s own beliefs from socially held expectations. The order of norm-elicitation and behavioural experiments may influence the norms and behaviours observed. Eliciting norms post-behaviour may lead to biases like self-serving ones^40^, while doing so pre-behaviour can cause people to overemphasise prevailing norms^41^, affecting their behaviour due to situational cues biases. To mitigate these biases, we randomly varied the order of eliciting people’s beliefs relative to their behavioural choices. On selected days (1, 5, 10, 14, 15, 19, 24), we also used the strategy method to elicit subjects’ conditional contributions based on different combinations of their group members’ behaviour and normative expectations (e.g. “How much do you contribute if most of the other people in your group contribute more than 50 and think that you should contribute more than 50”). When subjects respond to these questions, they do not know their group members’ behaviour nor their normative expectations, but, since we record these, we can implement their choice based on the contribution and expectations of others in their group. That is, this conditional contribution stage is incentivised and subjects know this (see Section S1.2 in the Supplementary Materials for details about the social norm belief elicitation method). On the final day of the experiment, we measure subjects’ preferences for punishing by allocating points to reduce the payoffs, at a 1:3 ratio, of a randomly selected other who contributed less than 50, 50, or more than 50. We also measure subjects’ expectations of how others behave in the punishment task. These expectations, again, were incentivised.

The treatments were a mixed within and between-subjects design. Specifically, both the severity (within-subjects) and the order of the risk (between-subjects) were manipulated: in the *High Low* treatment subjects first face a 90% risk (rounds 1–14) and then a 60% risk (rounds 15–28) while in the *Low High* treatment this is reversed.

In addition to the causal relationship between increased levels of risk and cooperation, the original study reported in Ref. ^18^ found that (1) empirical and normative expectations are positively associated with cooperation; (2) changing empirical and normative expectations changed cooperation; (3) punishment was targeted at low contributors and subjects anticipated this; (4) the stronger social norms that emerged under high risk made cooperative behaviour “sticky”, leading to a slower change in cooperation after a decrease in risk than the other way around (when risk increased, cooperative behaviour increased immediately); and (5) a specific combination of behavioural types emerged: empirical cooperators (increase cooperation in response to higher empirical expectations), normative cooperators (increase cooperation in response to higher normative expectations), social norm followers (increase cooperation in response to the combination of higher empirical and normative expectations), unconditional types (do not respond to empirical or normative expectations), and threshold-driven types (reduce their cooperation in response to higher empirical expectations). While compelling, these results rest entirely on risk that is experienced through monetary incentives: a higher probability of the collective loss event implies a higher probability of losing one’s earnings. Yet it remains unclear whether the same finding holds when the threat stems from a risk, due to the threat of Covid-19, that can physically harm individuals.

## Predictions

All hypotheses reported below were pre-registered at the Open Science Framework (https://osf.io/pufhm). Our first set of hypotheses concern outcomes that we *expected to change between the two waves*. Based on existing results that cooperation may increase after disasters, potentially including diseases, we anticipated that:

- Contributions will be higher in Wave 2 than in Wave 1 (Hypothesis 1).
- Groups are likelier to reach the threshold (i.e., to cooperate) in Wave 2 than in Wave 1 (Hypothesis 2).

Additionally, based on tightness-looseness theory, we predicted that:

- Average empirical expectations will be higher in Wave 2 than in Wave 1 (Hypothesis 3).
- Average normative expectations will be higher in Wave 2 than in Wave 1 (Hypothesis 4).
- The strength of group-level social norms will be higher in Wave 2 than in Wave 1 (Hypothesis 5).

By social norms strength, we mean here a combination of three factors. First, consistency: the extent to which people’s beliefs (empirical and normative expectations) within a group are consistent with each other’s. Second, accuracy: the degree to which these beliefs accurately capture reality. Empirical expectations should reflect the group's actual contributions and normative expectations should reflect the group’s personal normative beliefs. Third, specificity: the variance in the distribution of beliefs. The specific operationalization is taken from^18^ (see Supplementary Materials Section S1.3 for details).

We also anticipated that a number of other outcomes *should not change* between the two waves. In other words, some findings from Wave 1 should replicate in Wave 2. The first part of these expectations concern the behavioural types that were previously identified: empirical cooperators (increase cooperation in response to higher empirical expectations), normative cooperators (increase cooperation in response to higher normative expectations), social norm followers (increase cooperation in response to the combination of higher empirical and normative expectations), unconditional types (do not respond to empirical or normative expectations), and threshold-driven types (reduce their cooperation in response to higher empirical expectations). If we find these types again in Wave 2 then all types (but particularly normative cooperators and social norm followers) increase cooperation in response to higher normative expectations, and so we anticipate that:

- Increasing normative expectations generally enhances cooperation (Hypothesis 6a).

Instead, for increasing empirical expectations, empirical cooperators and social norm followers should increase their cooperation while threshold-driven types should reduce their contributions. Thus, we expected that:

- Increasing empirical expectations will decrease cooperation for some types of subjects and increase it for others. That is, the effect of empirical expectations on cooperation is more heterogeneous than the effect of normative expectations on cooperation (Hypothesis 6b).

Finally, the relationship between social norms and cooperation should not change either. Specifically, we should replicate that (1) empirical and normative expectation are positively associated with cooperation, (2) changing empirical and normative expectations changes cooperation, (3) punishment is targeted at low contributors and subjects anticipated this, and (4) stronger social norms developed under high risk make cooperative behaviour “sticky”, leading to a slower change in cooperation after a decrease in risk than after an increase in risk.

## Results

**Figure 2:**
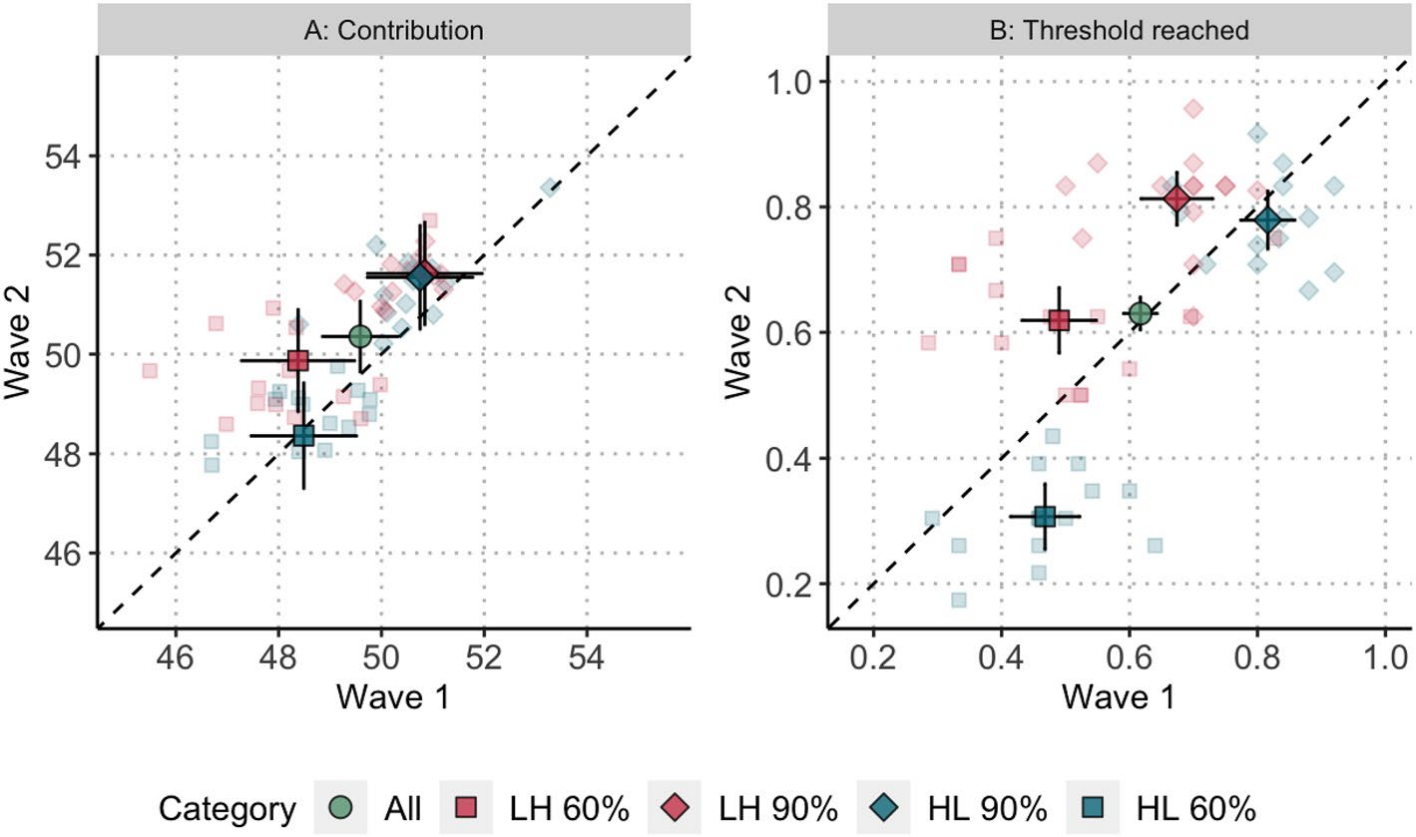
Scatter plots for contribution (individual level) and threshold reached (group level) in Wave 1 and Wave 2. *Note:* Green circle: All data; Blue square: Low High Treatment, 60% Risk; Blue diamond: Low High Treatment, 90% Risk; Red diamond: High Low Treatment, 90% Risk; Red square: High Low Treatment, 60% Risk. The area above the dashed diagonal indicates an increase in Wave 2 compared to Wave 1; the area below indicates a decrease. Dark solid shapes with confidence intervals represent the average predictor per category (All, LH60, LH90, HL90, HL60). Transparent shapes represent the average predictors per round.

**Figure 3:**
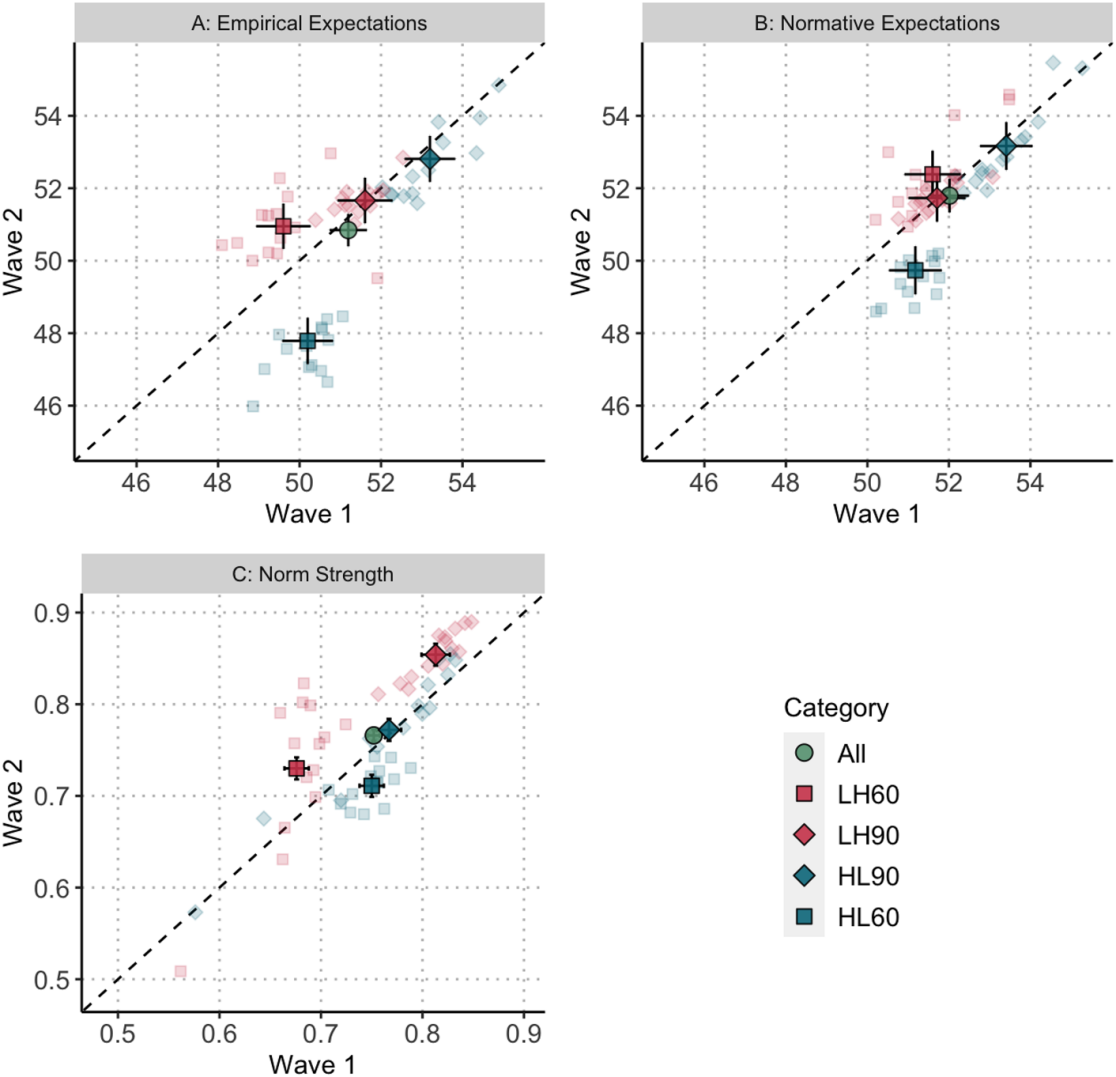
Scatter plots for empirical expectations, normative expectations (individual level), and norm strength (group level) in Wave 1 and Wave 2. *Note:* Green circle: All data; Blue square: Low High Treatment, 60% Risk; Blue diamond: Low High Treatment, 90% Risk; Red diamond: High Low Treatment, 90% Risk; Red square: High Low Treatment, 60% Risk. The area above the dashed diagonal indicates an increase in Wave 2 compared to Wave 1; the area below indicates a decrease. Dark solid shapes with confidence intervals represent the average predictor per category (All, LH60, LH90, HL90, HL60). Transparent shapes represent the average predictors per round.

We start by presenting the outcomes that we anticipated to change between the waves: contributions (H1), the probability that groups reach the threshold (H2), empirical expectations (H3), normative expectations (H4), and the strength of norms (H5). We then turn to the factors that we expected to remain comparable across the waves: behavioural types, the causal effect of empirical and normative expectations on contributions (H6a and H6b), and the relationship between social norms and cooperation. Table 1 qualitatively summarises the result for all preregistered hypotheses. Table 2 reports for Hypotheses 1–5 the predicted estimates in Wave 1 (column $\hat{y}_{Wave 1}$) and in Wave 2 (column $\hat{y}_{Wave2}$) and the regression coefficient for the wave difference. For each dependent variable, it first reports the overall effect of our preregistered hypotheses (*All*) and then proceeds with the results of exploratory analyses that compare the wave differences across treatments and risk levels (based on three-way interactions). Finally, Fig. 2 and 3 display the results of Hypotheses 1–5 visually in correlation plots that contrast the per-round correlation between Wave 1 (horizontal axis) and Wave 2 (vertical axis) for each round of the High Low (blue) and Low High treatment (red) for 90% risk (diamonds) and 60% risk (squares). Given this configuration, our hypotheses predict that the points on the figures should consistently lie above the 45^∘^ diagonal line. Detailed analyses including the effects of all control variables for all hypotheses are documented in the Supplementary Materials (Section S2). The Supplementary Materials also report the results of the replication analyses (Section S3) and of additional summary statistics and exploratory analyses (Section S4).

**Table 1:** Summary of our hypotheses and findings.

Hypotheses		Results
*Contribution and probability of reaching the threshold*		
H1	Contributions will be higher in Wave 2	Not supported
H2	Probability of reaching the threshold will be higher in Wave 2	Not supported
*Social norms: contents and strength*		
H3	Average empirical expectations will be higher in Wave 2	Not supported
H4	Average normative expectations will be higher in Wave 2	Not supported
H5	Strength of group-level social norms will be higher in Wave 2	Supported
*Behavioural types*		
H6a	Increasing normative expectations will increase contributions	Supported
H6b	Increasing empirical expectations will decrease contributions for some subjects and increase it for others	Supported

### Contribution and probability of reaching the threshold

We find no increase in contributions between Wave 1 and Wave 2 ($\hat{y}_{Wave1}=49.59$, $\hat{y}_{Wave2}=50.36$; $b_{All}= 0.768, p = 0.171$, 95% CI $[-0.33, 1.87]$) so do not find support for Hypothesis 1. To explore the lack of average differences, we check whether contributions varied across the waves according to risk level and treatment. We find that the predicted contributions in Wave 1 when the risk was 90% were greater than 50 in both treatments (Low High 90%: $\hat{y}_{Wave1}=50.84$, High Low 90%: $\hat{y}_{Wave1}=50.75$) implying that average contributions were already sufficiently high in Wave 1 under 90% risk to, in expectation, avert the possibility of a collective loss. Given the threshold at 300, any increase over 50 would result in inefficient cooperation (i.e., because resources are wasted). Consequently, and unsurprisingly, cooperation did not increase further in Wave 2 when risk was 90% (Low High 90%: $\hat{y}_{Wave2}=51.63$, High Low 90%: $\hat{y}_{Wave2}=51.55$; comparisons to Wave 1 n.s.). Under 60% risk of collective loss, average contributions in Wave 1 were always below 50 (Low High 60%: $\hat{y}_{Wave1}=48.38$, High Low 60%: $\hat{y}_{Wave1}=48.39$), thus allowing room for improvement. In Wave 2 contributions approached, but did not reach, 50 in Low High 60% of Wave 2 ($\hat{y}_{Wave2}=49.87$, comparison to Wave 1 n.s.), and failed to reach 50 in High Low 60% of Wave 2 ($\hat{y}_{Wave2}=48.37$, comparison to Wave 1 n.s.). Thus, the increase in contribution that we anticipated to see did not materialise: neither on average nor in the specific risk and treatment combinations.

**Result 1:** Contributions were not higher in Wave 2 than in Wave 1, neither on average nor broken down by treatment and risk level.

Similarly to contributions, we find no average increase in the probability of reaching the threshold across the waves (All: $\hat{y}_{Wave1}=0.62$, $\hat{y}_{Wave2}=0.63$; $b_{All}= 0.013, p = 0.470$, 95% CI $[-.02, .05]$) so do not find support for Hypothesis 2. We also check whether there are differences across waves according to risk level and treatment, and, unlike for contributions, find that the between-wave difference on the group level *does* depend upon the treatment. Subjects in Low High are significantly and substantially likelier to reach the threshold in Wave 2 than in Wave 1 ($b_{LowHigh}=0.132$, $p < 0.001$, 95% CI [0.082, 0.182]) and this result holds across both risk levels (Table 2). In High Low the opposite is true: subjects are less likely reach the threshold in Wave 2 ($b_{HighLow}=-\,0.98$, $p < 0.001$, 95% CI $[-\,0.147, -\,0.048]$). This difference is driven primarily by the decrease in the probability of reaching the threshold that happens following a decrease in risk. That is, in High Low 90 there is no difference between the waves (High Low 90: $\hat{y}_{Wave1}=0.82$, $\hat{y}_{Wave2}=0.78$; $b_{High Low 90}=-\,0.037, p = 0.235$, 95% CI $[-\,0.10, 0.02]$), but there is a significant and substantial decrease in High Low 60 ($\hat{y}_{Wave1}=0.47$, $\hat{y}_{Wave2}=0.31$; $b_{High Low 60}=-\,0.160, p <0.001$, 95% CI $[-\,0.23, -\,0.09]$). This is shown by the round contrasts in Fig. 2B that have most red shapes above the 45^∘^ diagonal line and most blue shapes below.

**Result 2:** Overall, groups were not likelier to reach the threshold in Wave 2 than in Wave 1. In Low High, reaching the threshold is higher in Wave 2, while in High Low reaching the threshold is lower in Wave 2.

**Table 2:** Summary of predicted outcomes in Wave 1, Wave 2, and their differences according to overall, treatment, and risk level based on marginal effects after multilevel regressions.

		$\hat{y}_{Wave1}$	$\hat{y}_{Wave2}$	*b*	*se*
**Contribution and reaching the threshold**					
*H1: Contribution*^1^	*All*	49.588	50.356	0.768	(0.560)
	Low High 60%^6^	48.377	49.869	1.492	(0.794)
	Low High 90%^6^	50.843	51.628	0.786	(0.800)
	High Low 90%^6^	50.750	51.550	0.800	(0.770)
	High Low 60%^6^	48.388	48.365	-0.023	(0.775)
*H2: Threshold reached*^2^	*All*	0.617	0.630	0.013	(0.018)
	Low High 60%	0.490	0.619	0.129***	(0.039)
	Low High 90%	0.674	0.813	0.139**	(0.035)
	High Low 90%	0.816	0.779	− 0.037	(0.031)
	High Low 60%	0.468	0.307	− 0.160***	(0.037)
**Social norms: contents and strength**					
*H3: Empirical expectations*^3^	*All*	51.196	50.845	-0.352	(0.340)
	Low High 60%	49.603	50.951	1.349**	(0.471)
	Low High 90%	51.607	51.662	0.055	(0.475)
	High Low 90%	53.202	52.810	− 0.391	(0.457)
	High Low 60%	50.198	47.788	− 2.419***	(0.460)
*H4: Normative expectations*^4^	*All*	52.016	51.796	− 0.220	(0.352)
	Low High 60%	51.599	52.385	0.786	(0.489)
	Low High 90%	51.709	51.730	0.021	(0.493)
	High Low 90%	53.408	53.166	− 0.242	(0.475)
	High Low 60%	51.177	49.736	− 1.441**	(0.477)
*H5: Norm strength*^5^	*All*	0.752	0.766	0.015**	(0.004)
	Low High 60%	0.676	0.730	0.054***	(0.009)
	Low High 90%	0.813	0.854	0.041***	(0.009)
	High Low 90%	0.767	0.772	0.005	(0.008)
	High Low 60%	0.750	0.711	− 0.038***	(0.009)

*Note:* ^1^ Multilevel OLS Regression of $N= 15181$ rounds nested in $N = 577$ individuals (see Table S2, Models 4–5); ^2^ Logistic regression of $N= 2588$ groups/rounds (see Table S3, Models 2–3); ^3^ Multilevel OLS Regression of $N= 15188$ rounds nested in $N = 577$ individuals (see Table S4, Models 4–5); ^4^ Multilevel OLS Regression of $N= 15183$ rounds nested in $N = 577$ individuals (see Table S5, Models 4–5); ^5^ OLS regression of $N= 2588$ groups/rounds (see Table S6, Models 2–3); Individual-level analyses of ^1^, ^3^, and ^4^ control for social value orientation, risk preferences, autism spectrum quotient, extraversion, agreeableness, conscientiousness, neuroticism, openness, age, gender, student, experience with experiments, and left-right political orientation. Analyses of ^1^ additionally control for empirical and normative expectations and personal normative belief; ^6^ Results based on exploratory analyses of three-way interactions between wave $\times$ treatment $\times$ disaster probability, see Model 5 in Tables S2, S4 & S5 and Model 3 in Tables S3 & S6.

### Social norms: contents and strength

We do not find the expected average increase of empirical expectations ($\hat{y}_{Wave1}=51.20$, $\hat{y}_{Wave 2}=50.85$; $b_{All}= -\,0.352, p = 0.301$, 95% CI $[- 1.02, 0.31]$) so do not find support for Hypothesis 3. Turning to the between-wave differences according to risk level and treatment, we find that, like for contributions, empirical expectations under 90% risk were already greater than 50 in Wave 1 of both treatments (Low High 90%: $\hat{y}_{Wave1}=51.61$, High Low 90%: $\hat{y}_{Wave1}=53.20$) making it unsurprising that Wave 2 empirical expectations are not significantly higher (Low High 90%: $\hat{y}_{Wave2}=51.66$, High Low 90%: $\hat{y}_{Wave2}=52.81$; comparisons to Wave 1 n.s.). Under 60% risk meanwhile, we find opposite differences according to treatment: an increase in empirical expectations in Low High ($\hat{y}_{Wave1}=49.60$, $\hat{y}_{Wave2}=50.95$; $b_{Low High 60}=1.349, p = 0.004$, 95% CI [0.43, 2.27]; Fig. 3A) and a decrease in empirical expectations in High Low ($\hat{y}_{Wave1}=50.20$, $\hat{y}_{Wave2}=47.79$; $b_{High Low 60}=-\,2.419, p < 0.001$, 95% CI $[-\,3.31, -\,1.51]$; Fig. 3A).

**Result 3:** Average empirical expectations were not higher in Wave 2 than in Wave 1. In Low High 60%, empirical expectations are higher in Wave 2 and in High Low 60% they are lower in Wave 2.

Turning to normative expectations, we also do not find the expected overall increase ($\hat{y}_{Wave1}=52.02$, $\hat{y}_{Wave2}=51.80$; $b_{All}= -\,0.220, p = 0.532$, 95% CI $[-\,0.91, 0.47]$) so do not find support for Hypothesis 4. Considering between wave differences by risk and treatment, we find a significant decrease in normative expectations in High Low 60% ($\hat{y}_{Wave1}=51.18$, $\hat{y}_{Wave2}=49.7$; $b_{High Low 60}=-\,1.44, p = 0.003$, 95% CI $[-2.38, -.51]$; Fig. 3B), which matches our result for empirical expectations. For all other combinations, we do not find significant differences (see Table 2).

**Result 4:** Normative expectations are not higher in Wave 2 than in Wave 1. In High Low 60% normative expectations are lower in Wave 2 than in Wave 1.

Concerning the strength of social norms (accuracy, consistency, and specificity of social expectations of group members), we find a statistically significant increase in Wave 2 relative to Wave 1 ($\hat{y}_{Wave1}=0.75$, $\hat{y}_{Wave2}=0.77$; $b_{All}= 0.015, p = 0.001$, 95% CIs [0.01, 0.02]). This supports Hypothesis 5. We find this increase in all three components of social norm strength: consistency across the social expectations of group members ($b_{All}=0.007$, $p=0.004$, 95% CIs [0.002, 0.012]), accuracy of social expectations ($b_{All}=0.005$, $p=0.001$, 95% CIs [0.002, 0.008]), and increased specificity in the range of acceptable behaviour and beliefs ($b_{All}=0.005$, $p=0.020$, 95% CIs [0.001, 0.009]; see Supplementary Tables S8–S9), although the increases are substantially minor and driven by the low variance on these indicators. Breaking down norm strength by risk and treatment, we find that social norm strength is higher in Wave 2 for both risk levels of the Low High treatment ($\hat{y}_{Wave1}=0.74$, $\hat{y}_{Wave2}=0.79$; $b_{Low High}=0.048$, $p < 0.001$, 95% CI [0.04, 0.06]; see the concentration of red shapes above the diagonal in Fig. 3C). For the High Low treatment, there is no between-wave difference when risk is 90% (H$\hat{y}_{Wave1}=0.77$, High Low 90: $\hat{y}_{Wave2}=0.77$; $b_{High Low 90}=0.005, p = 0.521$, 95% CI $[-\,0.01, 0.02]$; Fig. 3C) and a decrease in High Low 60% ($\hat{y}_{Wave1}=0.75$, $\hat{y}_{Wave2}=0.71$; $b_{High Low 60}=-0.038, p < 0.001$, 95% CI $[-\,0.05, -\,0.02]$; see the cluster of blue squares below the diagonal in Fig. 3C).

**Result 5:** Social norms are generally stronger in Wave 2 than in Wave 1. However, in High Low 60% social norms are weaker in Wave 2 than in Wave 1.

### Behavioural types

**Figure 4:**
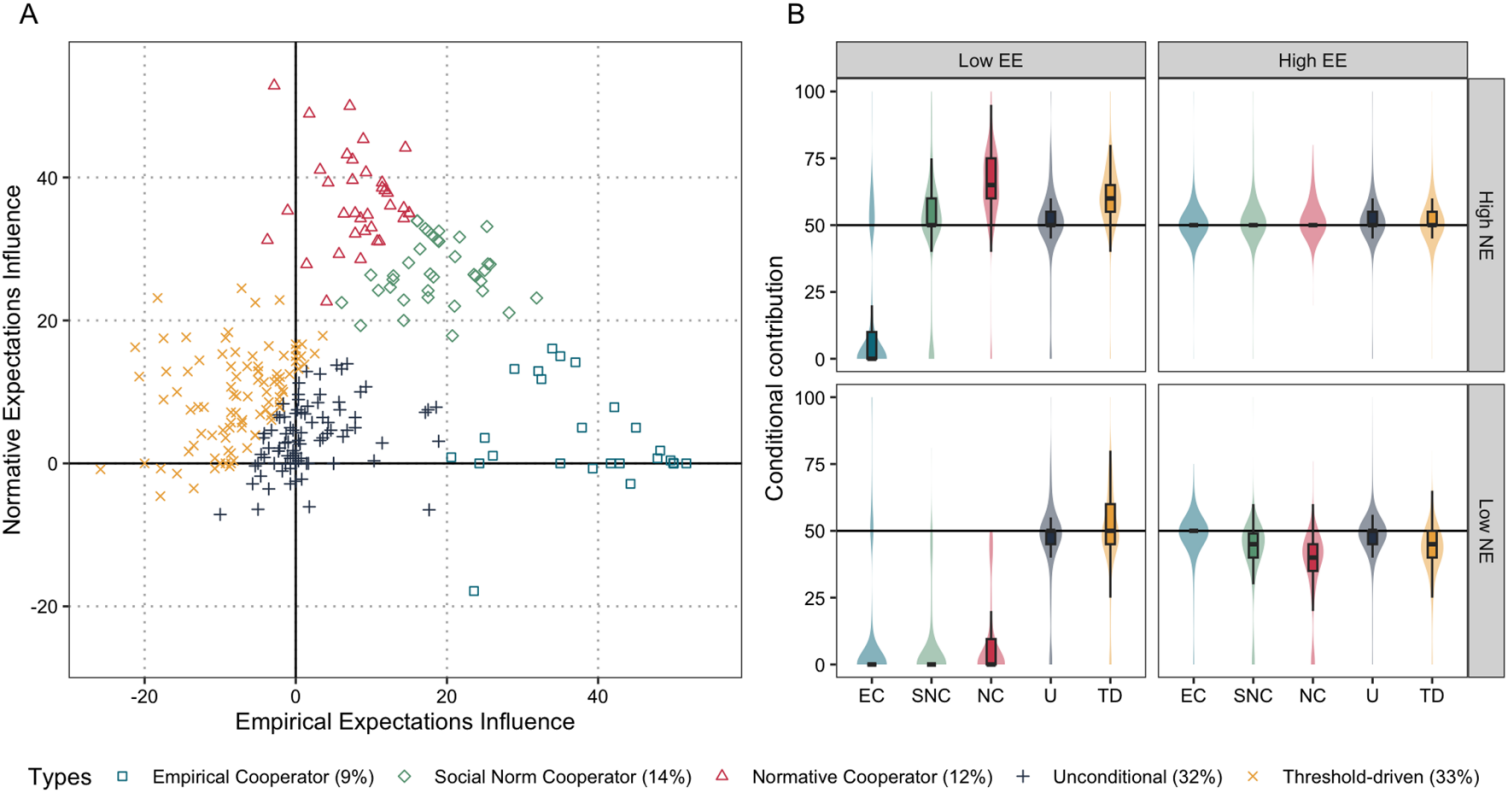
Behavioural type clusters and their conditional contributions in Wave 2. *Note:* EC, empirical cooperator; SNC, social norm cooperator; NC, normative cooperator; U, unconditional; TD, threshold-driven. Empirical (Normative) Expectations Influence is calculated by taking the difference in conditional contributions for High and Low EE (NE) averaged for when NE (EE) are high and low. Low EE (NE) refers to the hypothetical situation that the majority of the group contributes (thinks one should contribute) less than 50 points. High EE (NE) refers to the hypothetical situation that the majority of the group contributes (thinks one should contribute) at least 50 points.

We find the same behavioural profiles as in Wave 1 and in generally similar proportions (see Fig. 4A and B). Empirical cooperators (Wave 1: 11.6%, Wave 2: 9.0%) increase their contributions in response to higher empirical expectations, normative cooperators (Wave 1: 14.1%, Wave 2: 12.2%) increase their contributions when normative expectations are higher, social norm followers (Wave 1: 10.9%, Wave 2: 13.5%) increase their contributions in response to both higher empirical and higher normative expectations. We also find threshold-driven participants (Wave 1: 26.5%, Wave 2: 33.0%), in somewhat greater numbers than in Wave 1, who contribute around 50 points under all circumstances but slightly decrease contribution when others increase, and unconditional types (Wave 1: 37.0%, Wave 2: 32.2%), in somewhat smaller numbers than in Wave 1, who contribute around 50 points under all circumstances.

Consistent with Hypothesis 6a, we find that increasing normative expectations also increase the conditional contributions for all behavioural types irrespective of empirical expectations (see Fig. 4B and Supplementary Table S10). Some types increase their contributions a lot (e.g., Normative Cooperators increase their conditional contribution by 55 points for an increase in normative expectations if empirical expectations are low; $b=54.986$, $p < 0.001$, 95% CI [53.014, 56.96]), while others increase their contribution only slightly (e.g., a 4 point increase for Unconditional Types; $b=4.390$ $p < 0.001$, 95% CI [3.18, 5.60]).

Consistent with Hypothesis 6b, increasing empirical expectations does not increase contributions for all behavioural types. When normative expectations are low, most types respond to increasing empirical expectations by increasing their own contribution, but threshold-driven types instead reduce their contribution from 46.1 to 40.2 ($b=-\,5.914$, $p<0.001$, 95% CI$[-\,7.11, -\,4.72]$. When normative expectations are high, increasing empirical expectations has a negative effect not only on the contribution decisions of threshold-driven types ($b=-\,8.149$, $p < 0.001$, 95% CI $[-\,9.35, -\,6.95]$), but also of normative types ($b=-\,11.393$, $p < 0.001$, 95% CI $[-\,13.36, -\,9.42]$). However, this does not classify them uncooperative or as free-riders, for their contributions remain around 50. Rather, normative cooperators and threshold-driven cooperators compensate for their group members when empirical expectations are low (contributing on average 63 points and 60 points, respectively). When empirical expectations increase, this compensation is no longer needed and they reduce their contributions to 51 points (normative cooperators) and 49 points (threshold-driven types) on average.

**Result 6:** We identify the same five behavioural types as in Wave 1 and these respond homogenously to an increase in normative expectations, but show heterogeneity in their behavioural response to an increase in empirical expectations.

### Relationship between social norms and cooperation

Like in Wave 1, we find positive associations between empirical and normative expectations and contribution rates per round with one caveat: unlike in Wave 1, the positive association between normative expectations and contributions disappears after controlling for personal normative beliefs ($b=0.077$, $p=0.265$, 95% CIs $[-\,0.06, 0.21]$, see Supplementary Table S12).

We replicate the finding that contributions are causally and substantially influenced by empirical and normative expectations. When subjects are informed that the majority of group members contributes at least 50 points or believes one should contribute at least 50 points they significantly increase their contribution (see Supplementary Table S13 and Fig. S6). In Wave 1, the effect of normative expectations was stronger than that of empirical expectations. This effect is even more pronounced in Wave 2. The average contribution is 31 (95% CI [29.99, 32.51]) in response to low empirical and low normative expectations, it increases to 42 (95% CI [40.32, 42.84]) under high empirical and low normative expectations, and is further increases to 49 points when normative expectations are high—regardless of whether the high normative expectations are combined with low (95% CI [47.61, 50.13]) or high empirical expectations (95% CI [47.67, 50.20]).

A further indication of the presence of cooperative social norms comes from the replication that people punish low contributors more severely and expect others to do so as well (see Supplementary Tables S14 and S15). Low contributors ($<50$), irrespective of risk, are punished with a higher intensity (5.8 points, 95% CI [5.34, 6.26]) than those who contribute 50 points (2.27 points, 95% CI [1.90, 2.64]) or more (2.15 points, 95% CI [1.75, 2.54] and subjects expect low contributors to be punished with a higher intensity (6.58 points, 95% CI [6.17, 6.98]) than higher contributors (contribute 50: 3.55 points, 95% CI [3.13, 3.97]; contribute more than 50: 2.88 points, 95% CI [2.44, 3.33]).

Unexpectedly, we do not replicate the resilience of cooperative behaviour when moving from High to Low risk. In Wave 1 behaviour changed quickly after the change in risk in the Low High treatment, but norms exerted an inertia effect on behaviour in the High Low treatment. In Wave 2 we do not find this inertia effect for the High Low treatment. Instead, contributions in High Low drop immediately and significantly when risk changes, to levels even below the baseline of Low High (45.21 vs 50.04, $b=-\,4.833$, $p<0.001$, 95% CIs $[-\,7.23, -\,2.44]$; see Supplementary Tables S16 and S17 and Figure S11).

**Result 7:** We replicate most of the findings concerning the relationship between social expectations and cooperation. Exceptions are that cooperative behaviour is not resilient when moving from High to Low risk, and that normative expectations do not correlate with contributions after controlling for personal normative beliefs.

To summarise, our hypotheses about Covid-19 generating differences in contributions, probability of reaching the threshold, and social norm content (expectations) are not supported. Our findings do support the hypothesis that Covid-19 increased social norm strength. Moreover, the data support the hypotheses about the homogeneous effect of a manipulation of normative expectations and a heterogeneous effect of the manipulation of empirical expectations. Importantly, behavioural types are replicated, which speaks of the relevance of our classification, and most of the relationship between behaviour and norms is also replicated, except the resilience of the norm moving from high to low risk.

## Discussion

In this study we explored how cooperation and the associated social norms respond to collective disasters, with particular reference to disease threat like that posed by the Covid-19 pandemic. To this aim, we compared results of a collective-risk social dilemma experiment conducted before the pandemic (Wave 1, 2018) to the results from a replication of the same experiment that we collected near the height of the first Covid-19 pandemic wave (Wave 2, 2020).

On the basis of empirical evidence and theory, we predicted an increase of cooperative behaviours and social norms. Contrary to our expectations, we did not find this overall increase in cooperation and social norms in Wave 2. One reason for this result might be the very nature of our game, in which cooperation over an average of 50 is not efficient and therefore participants on average do not need to increase their efforts. Indeed, exploratory analyses reveal that the results depended on treatment and risk. In Wave 2, groups that start with a low disaster probability (60%) have stronger norms and are also significantly better at cooperating to reach the threshold compared to Wave 1. This advantage is revealed initially when groups face a low risk, but such improvement over Wave 1 continues to exist when risk increases to (90%). Such effect may indeed indicate that shared acquaintance with the pandemic might affect the alignment of social expectations and thereby increase the ability to coordinate with others: experience with the Covid-19 threat might have taught people to better estimate how others behave and how one should behave to avoid the disaster, even if the risk of that disaster is smaller. While in Wave 1 participants who started with a high disaster risk of 90% managed to cooperate and create strong social norms from the start more often than those starting in the low disaster risk; with the experience of the pandemic also participants that started with a lower, 60% risk mostly managed to cooperate.

Yet it remains unclear why social norms are weaker and why the ability to coordinate decreases in the low risk stage of the other, High Low treatment. Specifically, groups that start with a high disaster probability (90%) perform worse in Wave 2 compared to Wave 1 as cooperation decreases and social norms get eroded compared to Wave 1 when risk decreases to (60%) after 14 rounds. This pattern may suggest that during the Covid-19 pandemic, subjects might have realised that the need to cooperate—e.g., keeping social distance or wearing masks—is conditional on risk. When risk decreases, the possibility of getting infected becomes lower, and as such the need to cooperate. This might have translated into reduced (expectations about) cooperation in our experimental setting. This is a speculative explanation, though, that could not be tested with the available data. Further research based on data of (changes in) individual risk perception would be needed to better disentangle how norms and behaviour change in response to repeated recurrences of risk.

Even if some of our hypotheses were not confirmed, our findings have interesting theoretical implications. Contrary to our expectations grounded in the tightness-loose theory of cultures^17^, we do not have evidence that the global threat posed by the Covid-19 pandemic was sufficient to create a rapid general increase in cooperation and the underlying social norms. Evidence of the predicted increase is available only under specific scope conditions—suggesting, for instance, that the pandemic improved recognition of the potential disaster and the ability to cooperate when a risk is introduced, but that this is temporary and may break down as soon as the risk becomes less severe. Yet, this pattern of results may still be consistent with the broader theoretical stance. Indeed one possible explanation for our results could be that more time is needed for effects on norms and behaviour to emerge. Our study has been conducted in the first months of the pandemic and social behaviour and norms follow complex patterns that might differ between the short and long run. Another possibility is that different threats may strengthen different social norms, in particular those that are most relevant to overcome the specific threats faced. For example, pandemic threats might increase norms of hygiene, while hurricanes make stronger norms of helping. The cooperative task in the experimental study might have been perceived by subjects as not pertinent or too abstract to be relevant for the threat posed by the pandemic. This would be consistent with a cross-country study conducted at the beginning of the Covid-19 pandemic which found that in the short term, norms are largely stable to pandemic threats except for those norms that are perceived to be directly relevant to dealing with the collective threat, in particular hand washing norms^28^. Still, other research carried out comparing behaviours in countries under similar circumstances^19^ showed a slightly significant increase in trust among infected people. While the authors conjectured that this could be due to receiving more cooperative behaviour when sick, we cannot make a clear connection with our results because we do not have separate data on infected versus non infected people.

Regardless, replicating the findings of our previous work^18^, this study provides evidence that under high risk (90% probability treatment), social norms become tighter and promote cooperation. Moreover, experience with the pandemic made participants more cooperative under 60% already in the Low High treatment. This is consistent with the core hypothesis of the tightness-looseness theory^17^. Recognising that social norms are strong drivers of cooperation is relevant to understanding how to design interventions to promote long-lasting cooperation in collective action problems characterised by risk. As the experience of the Covid-19 pandemic has made clear, in the mitigation of collective crises the use of messages that emphasise their risk and severity might not be effective as they can be paralyzing for people. If global challenges are perceived to be too big to be handled individually, this can evoke anxiety that might cause in people feelings of powerlessness and the impression that there’s nothing we can do about it.^42,43^ Instead, interventions relying on social norms (or a combination of risk and norms) can appeal to a collective effort and to the fact that we can make it if we all act together.

On a fundamental level, our results also show how important social norms are to cooperation. Indeed, in the manipulations of expectations, both normative and empirical expectations substantially affect one’s own contributions. This reinforces a key result from Szekely et al.^18^. Moreover, Wave 2 convincingly replicated the existence of behavioural types. Not only can participants be classified in the same groups, but even the percentage of each type in the population is approximately the same. This finding, along with related results in other experiments^44,45^, stresses the importance of recognizing different types of behavioural responses, for instance in the context of policy implementation.

Needless to say, our study presents several limitations. First, the cooperative behaviour in the task might be perceived as too abstract to be relevant for the specific risk posed by the Covid-19 pandemic. This might makes it difficult to study whether a change in a real collective risk, such as the one posed by the pandemic of Covid-19, is associated with changes in cooperative behaviour and social norms sustaining it. Second, we analyze short-time effects. Wave 2 has been conducted in the first months of the Covid-19 pandemic and the time frame might be too short to capture changes in social norms and behaviour. Third, our design cannot entirely identify the causal effect of a variation in disease threat severity on the strength of social norms and cooperation. We cannot exclude that changes in norm strength and cooperation might have happened over time independent from the pandemic or other confounds nor did our design allow for a comparison between (pandemic-related) risk perceptions across waves. Fourth, in contrast with a one-shot design, the use of a paradigm with repeated but anonymous encounters allows the formation of group-level norms within the confine of an experimental setting. However for this very reason, this design choice may also reduce the identification of broader social norms that subjects bring to the lab from their everyday experience. Finally, we have employed a standard subject pool of university students. This should not be an issue for our between-waves comparison, since samples are similar between the waves, but it needs to be considered when generalizing our findings to the broader population.

## Methods

### Experiment

The replication experiment was conducted in the same way as the original experiment. On the first day, subjects completed the Big Five personality questionnaire, the Social Value Orientation slider measure, the Autism Spectrum questionnaire, and a risk preference elicitation task. On days 2–29, they participated in 28 rounds (one round per day) of the collective-risk social dilemma. At the end of the experiment (day 30), subjects' punishment behaviour and their expectations concerning punishment were elicited and they answered a short questionnaire on sociodemographics. The experiment was programmed in oTree. All interactions took place anonymously on computers or phones.

On days 2–29, subjects were randomly allocated into groups of six each round. Every round, they received an endowment of 100 points and had to decide how much of their endowment to contribute to a collective fund. If a threshold amount (300 points) was reached, subjects kept as that round’s earnings the share of their endowment not contributed to the collective fund. If the threshold was not reached, with some probability *p* they lost their entire endowment, while with probability $1-p$ they kept what they did not contribute to the collective fund. Subjects that participated in the treatment High Low faced a 90% risk in rounds 1–14 and a 60% risk in rounds 15–28. Subjects in the treatment Low High first faced the 60% risk and after 14 days switched to the 90% risk.

Contribution decisions were made simultaneously and anonymously. To study whether social norms were formed, each round we elicited subjects’ Personal Normative Beliefs (PNB), Empirical Expectations (EE), and Normative Expectations (NE) randomly either before or after they made their contribution decision. The EE decisions were incentivised by comparing them against the group members’ contributions; the NE decisions were incentivised by comparing them against the group members’ PNBs. At the end of each round subjects were informed about the contribution decisions of the group members. In rounds 1, 5, 10, 14, 15, 19, 24, 28, subjects were presented with additional conditional contribution questions in which they were asked for their hypothetical contributions in case the majority of their group members would contribute [at least 50 points/less than 50 points] and believed one should contribute [at least 50 points/less than 50 points]. These questions were incentivised by paying the subjects based on their answer to the EE and NE combination that was realised in the subject’s group.

### Data

The 286 subjects who participated in Wave 1 and the 293 subjects that participated in Wave 2 were all recruited from the IBSEN subject pool (http://www.ibsen-h2020.eu). Subjects who participated in Wave 1 could not participate in Wave 2. All subjects are Spanish or residents in Spain. 55% of the subjects were female in both waves. The subjects of both waves differ significantly with respect to their average age (30 in Wave 1, 24 in Wave 2), the percentage of students (50% vs 83%), and the percentage that participated in at least one other experiment before (43% vs 73%). There are no differences with respect to their political orientation, social value orientation, risk preferences, autism spectrum quotient, and Big Five characteristics (See Table S18 in the Supplementary Materials).

Subjects earned an average of €27.67 including a €5 show-up fee and a 10× multiplier to the earnings of one randomly selected subject in both waves to minimise the risk of dropout (to a maximum of €200). The data collection of Wave 1 occurred in two separate sessions. The High Low treatment started on June 4 2018 for 30 days, while the Low High treatment was conducted starting on September 18 2018. For Wave 2, both treatments were run simultaneously to avoid changes in the pandemic influencing the outcomes. The data was collected for 30 days starting June 8 2020. Subjects that failed to make a decision on four different days were excluded from the experiment. In total, 23 dropped out or were excluded in Wave 1 (3 in High Low, 20 in Low High) and 18 in Wave 2 (9 per treatment) (See Table S19 in the Supplementary Materials). The study received institutional ethical approval from the ethics board of the Institute of Cognitive Sciences and Technologies (Italian National Research Council) and informed consent was obtained from all subjects. All research was conducted in line with the relevant regulations.

### Statistical methods

All hypotheses and the analysis plan were preregistered on the Open Science Framework (https://doi.org/10.17605/OSF.IO/PUFHM). Hypotheses 1–5 test the Wave 2–Wave 1 difference. Individual-level hypotheses H1 (contribution), H3 (empirical expectations), and H4 (normative expectations) were tested using multilevel OLS regressions that also controlled for Social Value Orientation, risk aversion, Autism Spectrum Quotient, Big Five characteristics, age, gender, political orientation, whether the subject was a student, and whether they had experience with other experimental studies. H1 additionally controlled for empirical expectations, normative expectations, and personal normative beliefs. Hypotheses H2 (threshold reached) and H5 (norm strength) are tested on the group-level. For H2 we used multilevel logistic regressions to estimate the probability that groups reach the threshold of 300 points. To test H5 we run multilevel OLS regressions on an index of social norm strength as developed in^18^ that was based on three criteria: (1) agreement between expectations of group members (consistency), (2) whether expectations correctly predict the behaviour and personal normative beliefs of others (accuracy), and (3) how specific the norm is concerning the range of acceptable behaviours (specificity). (see Supplementary Information Section 1.3 for precise definitions and the measurement scale).

In addition to the preregistered analyses of overall wave differences we explored why Wave 2 did not bring the anticipated increases in our dependent variables. We ran exploratory analyses to test whether the effect of Covid-19 differs per treatment (Low High or High Low) and per risk-level (60% or 90%) by testing the effect of Wave 2 in interaction with treatment, risk level, and treatment $\times$ risk level. To facilitate the interpretation of the result of these three-way interactions we calculate the marginal effect on the dependent variable for each combination.

To test H6a and H6b, we first identify behavioural types using *k*-means clustering of subjects based on their responsiveness to the manipulated empirical and normative expectations (see Supplementary Information Section 1.4). We then ran OLS regressions on conditional contributions of Wave 2 to test whether contributions were different for the different manipulations of the expectations (Low EE/Low NE, Low EE/High NE, High EE/Low EE, and High EE/High NE) and their interactions with each of the behavioural types.

### Supplementary Information

Supplementary Information.

## Data Availability

The experiment code, the data, and the scripts to reproduce the analyses and figures are stored at the Open Science Framework (https://osf.io/xb8a7).
