# Supplementary material for: Assessing the effects of pandemic risk on cooperation and social norms using a before-after Covid-19 comparison in two long-term experiments
Source: Sci Rep. 2024 Feb 9;14:3356. doi: 10.1038/s41598-024-53427-z (PMC10858192; doi:10.1038/s41598-024-53427-z)
Supplement: Supplementary file 1 — Supplementary Information. [file 41598_2024_53427_MOESM1_ESM.pdf]

Supplementary Materials to "Assessing the effects of pandemic risk on cooperation and social norms using a before-after Covid-19 comparison in two long-term experiments"

Eva Vriens[1,2,*], Aron Szekely[3], Francesca Lipari[4], Alberto Antonioni[4], Angel Sánchez[4,5], Luca Tummolini[1,2], and Giulia Andrighetto[1,2,6]

[1]Institute of Cognitive Sciences and Technologies, Italian National Research Council, Rome, Italy
[2]Institute for Futures Studies, Stockholm, Sweden
[3]Collegio Carlo Alberto, Turin, Italy
[4]Grupo Interdisciplinar de Sistemas Complejos (GISC), Departamento de Matemáticas, Universidad Carlos III de Madrid, Leganés, Spain
[5]Instituto de Biocomputación y Física de Sistemas Complejos (BIFI), Universidad de Zaragoza, Zaragoza, Spain
[6]Malardalens University, Vasteras, Sweden
[*]eva.vriens@istc.cnr.it

January 9, 2024

Contents

1 Study design **4**
- 1.1 Experiment instructions . . . 4
- 1.2 Social norm belief elicitation method . . . 13
- 1.3 Social norms strength definition and measurement . . . 15
  - 1.3.1 Consistency . . . 15
  - 1.3.2 Accuracy . . . 16
  - 1.3.3 Specificity . . . 16
- 1.4 Analysis of behavioral typology . . . 18

2 Effect of Covid-19 hypotheses **19**
- 2.1 H1: Covid-19 increases the average contribution . . . 20
  - 2.1.1 Analytical strategy . . . 20
  - 2.1.2 Results . . . 20
- 2.2 H2: Covid-19 increases the likelihood of groups reaching the threshold . . . 23
  - 2.2.1 Analytical strategy . . . 23
  - 2.2.2 Results . . . 23
- 2.3 H3 & 4: Covid-19 increases EE and NE . . . 25
  - 2.3.1 Analytical strategy . . . 25
  - 2.3.2 Results . . . 25
- 2.4 H5: Covid-19 increases norm strength . . . 29

2.4.1 Analytical strategy 29
2.4.2 Results 29
2.5 H6: Effects of normative and empirical expectations 33
2.5.1 Analytical strategy 33
2.5.2 Results 33

3 Replication of original hypotheses **36**
3.1 H1: EE and NE as predictors of contribution 37
3.2 H2: Contribution according to manipulated expectations 39
3.3 H3: Punishment behavior and beliefs 42
3.4 H4: Faster behavioral change in Low to High Treatment 43

4 Additional summary statistics and analyses **45**
4.1 Summary statistics 45
4.2 Summary statistics dropout 47
4.3 Individual predictors of Empirical Expectations Influence and Normative Expectations Influence 48
4.4 Dynamics in social norm strength and its components 49
4.5 Dynamics in contributions, beliefs, and social expectations 51
4.6 Groups reaching threshold according to social norm strength 52
4.7 Individual predictors of round 14 to round 15 contribution change 53

List of Tables

S1 Summary of hypotheses and results for the replication study (W2). 19
S2 Average Contribution by Wave, Treatment, and Risk Level 22
S3 Likelihood of reaching the threshold by Wave, Treatment, and Risk Level 23
S4 Average Empirical Expectations by Wave, Treatment, and Risk Level 26
S5 Average Normative Expectations by Wave, Treatment, and Risk Level 27
S6 Social Norm Strength by Wave, Treatment, and Risk Level 30
S7 Social Norm Accuracy by Wave, Treatment, and Risk Level 31
S8 Social Norm Consistency by Wave, Treatment, and Risk Level 31
S9 Social Norm Specificity by Wave, Treatment, and Risk Level 32
S10 Conditional contributions per behavioral type in Wave 2 35
S11 Summary of results of original (W1) and replication study (W2). 36
S12 Predictors of contribution (W2). 38
S13 Contribution according to manipulated expectations (W2). 39
S14 Punishing points allocated by collective risk probability and treatment 42
S15 Beliefs about punishing points by collective risk probability and treatment 42
S16 Comparison test of cooperation level for round 15 among treatments 43
S17 Dynamics of cooperation after the change in risk by treatment (round 15-28) . . 43
S18 Summary statistics by treatment and overall, round 1 and 28 45
S19 Dropout by the end of the experiment 47
S20 Individual predictors of EE Influence and NE Influence 48
S21 Groups reaching the threshold according to norm strength 52
S22 Individual predictors of contribution change in Wave 2. 53

List of Figures

S1 Average Contribution by Wave, Treatment, and Risk Level 21
S2 Probability of reaching the threshold by Wave, Treatment, and Risk Level 24
S3 Average Empirical and Normative Expectations by Wave, Treatment, and Risk Level 28
S4 Social Norm Strength by Wave, Treatment, and Risk Level 30
S5 Conditional contributions and behavioral types in Wave 2 33
S6 Violin plot of distribution of conditional contributions according to EE and NE . 40
S7 Violin plot of conditional contributions according to EE and NE by Wave and Treatment 41
S8 Social norm and contribution dynamics in Wave 2 49
S9 Social norm strength components by round and broken down by treatment . . . 50
S10 Contributions and expectations by round according to treatment 51
S11 Proportion of groups reaching threshold by social norm strength and wave 52

1 Study design

1.1 Experiment instructions

In this section we include the translation from Spanish of the exact instructions form that participants received for the two experimental treatments, named High Low (HL) and Low High (LH). A resumed version of the instructions was always available to participants during the entire experiment. The Spanish version of the instructions can be made available upon request. The cartoon images shown on pp. 8,9 were included in the instructions for subjects. The instructions for the Big Five, Risk preference elicitation, Autism spectrum measurement, demographic questionnaire, and Social Value Orientation are not reported here and can be requested from the authors. *[Comments about experimental protocol that were not shown to participants will be shown below in brackets.]*

Welcome page

Welcome and thanks for participating in this experiment. The experiment consists in seven sections. You will begin by doing the first five sections, which will take approximately 20 minutes. You can do it right after reading these instructions. These sections are mandatory and you will be automatically excluded if you will not complete them. You have until tomorrow at **10 AM (Madrid time)** to complete these first five sections. After that, you will be moved to the sixth section. You can take the first decision during this section tomorrow. Then, you will be participating in a round of the sixth section all the other days. You will have until **10 AM (Madrid time)** of the following day to make your decisions for that day. Your decisions during this section will not take more than few minutes per day. The last section, the seventh, will take few minutes to be completed.

Important rules

- Your participation is voluntary and you can leave the experiment at any time. However, in that case, you will not receive any payment.
- You are asked to not communicate with other participants and, in general, to make your decision in an independent manner. You may not share your participation link with anyone.
- Your answers will be kept confidential.
- During the entire experiment there will be not any form of deception.

Payment

You will be paid at the end of the experiment. Your payment will be the sum of all your earnings during each section. For each **30 points you gain you receive 1 EUR**, rounded to the closest integer number. Your earnings will depend on your decisions and the decisions of other participants, and partially from some randomness. Your payment will be made through PayPal, in a manner that other participants will be not able to know how much you gained. Additional instructions on how you can make extra points will follow.

If you do not complete the decisions of today or some decisions during the sixth section, you will be **automatically and permanently** excluded from the experiment. **If you will be excluded, you will not be paid.**

At the end of the experiment, one participant among those who finish the experiment will be randomly selected to gain an extra bonus. The bonus means that all the earnings of the selected participant will be multiplied by 10. We guarantee that **the randomly selected person will gain at least 100 EUR and up to 200 EUR.**

Additional information

Please remember that once you click on the "Next" button below you cannot come back to this screen. Please always read carefully all experimental instructions. We will always show you a compact version of the instructions. If you have any doubt during the experiment, please get in touch with us (cnr.ibsen@gmail.com). Please click on the "Next" button.

[After this page participants perform Big 5 (section 1), SVO test (section 2), Autism spectrum (section 3), demographic questions (section 4) and risk preference test (section 5)]

End of day 1 page

You have completed all decisions of today. Come back tomorrow at **10 AM (Madrid time)**.

Instructions of section 6 page

You are going to interact with other **5 participants**. Their identities will remain unknown for the entire experiment. At each round, you will receive a round endowment of **100 points** and you can choose how many points you want to invest in a common pot.

If your group accumulates **300 points** in the common pot all of you will save the points you did not invest.

If the accumulated investments do not reach the threshold, the system will choose a random number between 1 and 10. If this number is less than or equal to **9** (HL treatment) **6** (LH treatment) **you all will lose all points** and you will not gain any point from that round.

On the other hand, if this number is larger than **9** (HL treatment) **6** (LH treatment) all of you will save the points you did not invest.

In other words, if the points in the common pot during a round are less than 300, there is a probability of **90%** (HL treatment) **60%** (LH treatment) that you lose all points for that round. To summarize, there are three possible outcomes for each round. If your group accumulates:

- **at least 300 points**, all participants in the group **save the points they did not invest**.
- **less than 300 points** and the system draws a number less than or equal to **9** (HL treatment) **6** (LH treatment), all participants in the group **lose all the points for that round**.
- **less than 300 points** and the system draws a number larger than **9** (HL treatment) **6** (LH treatment), all participants in the group **save the points they did not invest**.

You will take part in 28 rounds of this section of the experiment. At the beginning of each round, you will be randomly grouped with other 5 participants in the experiment.

At the end of the experiment the system will randomly select four rounds. One for each week of the experiment. The amount you have gained during those four rounds will be added to your final payment. Each round is independent from each other in terms of your earnings.

During these rounds there will be some additional questions that will allow you to gain extra points. In some rounds you will be asked to make your decisions in a different framework, so please pay attention to all the instructions. Every change will be underlined during the experiment.

At the end of the experiment, a participant will be randomly selected. The points gained by the selected person will be multiplied by 10. This means that **the selected person can gain up to 200 EUR (with a guaranteed gained of 100 EUR)**.

Important note: if you do not take a decision for more than 3 days you will be automatically excluded from the experiment and you will not gain anything from it. **If a participant does not take a decision during a round, a random decision chosen from another person in the same group will be automatically implemented**.

These instructions will be available during the entire experiment at the end of each screen.

Examples page

Example 1
In the first round you have been grouped with other 5 people. Each participant receives 100 points. You contributed 0 points and the others contribute 50 points each. As a consequence, the total amount invested in the common pot is 250 points (= 0 points + 5 x 50 points), which does not reach the needed 300 points.

The system draws a random number from 1 and 10. The number is 2, this means that all of you gain no points from this round.

Example 2
In the second round you have been grouped with other 5 people. Each participant receives 100 points. You contributed 75 points and the others contribute 75 points each. As a consequence, the total amount invested in the common pot is 450 points (= 75 points + 5 x 75 points), which does reach the needed 300 points.

As a consequence, you gain 25 points from the saved points you did not contribute (= 100 points - 75 points), as your groupmates.

Example 3
In the third round you have been grouped with other 5 people. Each participant receives 100 points. As in the first round, you contributed 0 points and the others contribute 50 points each. As a consequence, the total amount invested in the common pot is 250 points (= 0 points + 5 x 50 points), which does not reach the needed 300 points.

The system draws a random number from 1 and 10. The number is 10, this means that all of you gain the points you did not contribute. In this case, you gain from this round 100 points.

Questions page

Please answer the questions below. They are designed to help you better understand section 6. Your answers will not have any consequences for your payments. WE remind you that you can see the complete instructions for this section in a box at the bottom of this page (move the page up if necessary).

Question 1
Your group contributed in total 350 points in this round. What happens?

- The group contributed enough points and all group members gain saved money.
- All group members lose all points.
- All group members lose all point with probability of **90%** (HL treatment) **60%** (LH treatment).

Question 2
Your group contributed in total 150 points in this round. What happens?

- The group contributed enough points and all group members gain saved money.
- All group members lose all points.
- All group members lose all point with probability of **90%** (HL treatment) **60%** (LH treatment).

Question 3
You contribute 50 points in this round, all your group members contributed 250 points. How much do you gain in this round?

- 0
- 50
- 70

Question 4
You participate in this experiment always with the same 5 people in each round. [True/False]

Question 5
What happens if you miss three or more decisions during the experiment?

- Nothing
- You will be excluded and not paid

Answers page

You have correctly answered to x out of 5 questions. Here they follow correct answers. Section 6 will begin after this page. *[Participants are shown correct answers.]*

First round page (days 2-29)

You are going to begin the round X out of 28. This means that you are playing the day (X+1) out of the experiment. **You have been randomly grouped with other 5 people.**

Personal Normative Belief page (days 2-29)

How many points should a person in your group, including yourself, contribute?

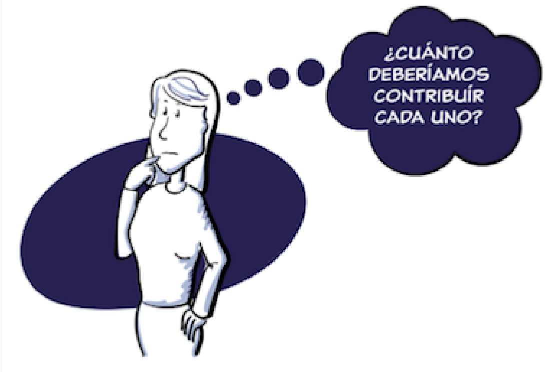

Empirical Expectations page (days 2-29)

You now have the opportunity to earn additional points. At the end of the experiment, you will be told if you have earned these points.

How many points will the other 5 people in your group spend?

Use the boxes below to indicate the contributions **you think the other people in your group will make**. Put the highest value in the box at the top and then rank the contributions in descending order. You can enter the same value for several people. In that case the order for those people does not matter. We will rank the contribution of the other people in your group in this round and compare each of them with your answers. For each answer you believe to be completely correct you will get 5 points. This means you can earn a maximum of 25 points. The less accurate your answer is, the less points you will receive. If your answer differs from the actual values by more than 5 points then you receive 0 points for those answers.

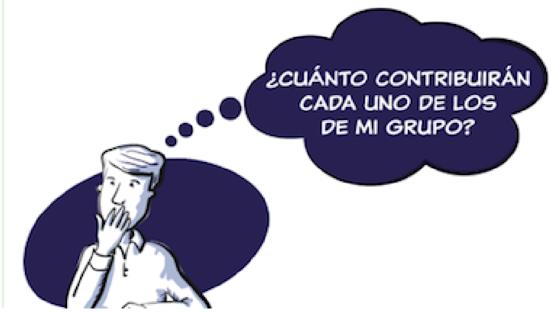

Normative Expectations page (days 2-29)

You now have the opportunity to earn additional points. At the end of the experiment, you will be told if you have earned these points.

How many points will the other 5 people in your group think you all should spend?

Use the boxes below to indicate **how many points each of the people in your group think each of you should contribute**. Put the highest value in the box at the top and then rank the contributions in descending order. You can enter the same value for several people. In that case the order for those people does not matter. We will rank the responses to the previous question ('How many points should a person in your group, including yourself, contribute?') of the other people in your group in this round and compare each of them to your responses. For each answer you believe to be completely correct you will get 5 points. This means that you can earn a maximum of 25 points. The less accurate your answer is, the less points you will receive. If your answer differs from the actual values by more than 5 points, then you receive 0 points for these answers.

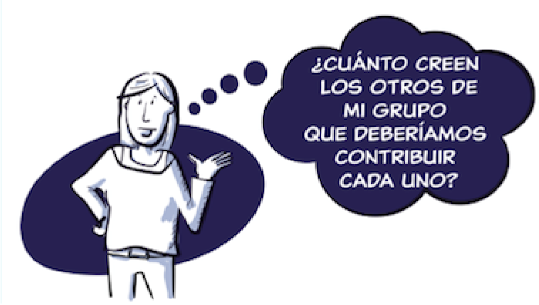

Contribution page (days 2-29)

How many points do you want to invest?

Conditional scenario page

[for days 2/5/8/9/12/15/16/19/22/23/26/29 only]
In this round, we ask you to make an additional decision to earn extra points. We ask you what you would contribute in different scenarios. For each of the four situations presented, indicate what you would do. Since we will record the responses of each group member as well as what they believe, we will implement your choice to the case that it actually happened.

Specifically, you have all indicated to us how much you think should be invested. From here, we select which of your decisions are carried out. For example, if the majority of your group members put in at least 50 points and believe that you all should spend at least 50 points, then we will use the answer you have given us in the first row to calculate the group's contribution for this additional round.

How many points will you invest if:

- the majority of your group members put in **at least 50 points** and believe that you all should spend **at least 50 points**.
- the majority of your group members put in **less than 50 points** and believe that you all should spend **at least 50 points**.
- the majority of your group members put in **at least 50 points** and believe that you all should spend **less than 50 points**.
- the majority of your group members put in **less than 50 points** and believe that you all should spend **less than 50 points**.

End of the round page (days 2-29)

You have completed all decisions of today. Come back tomorrow at **10 AM (Madrid time)**.

Results of the previous round page (days 2-29)

The previous round has finished. You have begun with **100 points. You invested XX points and your group contributed a total of YY points**. The complete list of contributions follows below. Contributions are listed in a random order.

[Option 1: Threshold achieved]
This means that your group accumulated enough points, and you all save the points you did not spend in this round.

[Option 2: Threshold not achieved, and points saved]
This means that your group did not accumulate enough points. The system randomly took the number 10 and you all saved the points you did not spend in this round.

[Option 3: Threshold not achieved, and points lost]
This means that your group did not accumulate enough points. The system randomly took the number 1 and you all loose the points you did not spend in this round.

Results of the previous round page: conditional scenarios

(days 3/6/9/10/13/16/17/20/23/24/27/30 only)
You have begun with **100 points. You invested XX points and your group contributed a total of YY points**. The complete list of contributions is the same of the above one.
[Note that the random number for conditional scenarios is not necessarily the same]

[Option 1: Threshold achieved]
This means that your group accumulated enough points, and you all save the points you did not spend in this round.

[Option 2: Threshold not achieved, and points saved]
This means that your group did not accumulate enough points. The system randomly took the number 10 and you all saved the points you did not spend in this round.

[Option 3: Threshold not achieved, and points lost]
This means that your group did not accumulate enough points. The system randomly took the number 1 and you all loose the points you did not spend in this round.

Your total earnings in this round are ZZ points.

15th round page (also shown in following 3 rounds)

There is a change in the experiment. From this round until the end of the experiment, there is a change. **The probability that you all lose all your points if the common pot does not reach 300 points is now of 60%** (LH treatment) **90%** (HL treatment). All other settings remain the same as before. As before, if your group does not reach the needed points the system will randomly draw a number from 1 to 10 and if this number is equal to or less than **6** (LH treatment), **9** (HL treatment). If this number is larger than **6** (LH treatment), **9** (HL treatment) you keep all saved points.

After 28th round

You have been paired with another randomly chosen participant. You will not know the identity of the other person and the other person will not know yours.

All participants, including yourself, receive 30 points. You are now asked to take decisions in these hypothetical situations that will be applied to the person with whom you are paired and according to the level of contribution in the last round that person made. You can use up to ten points in this round. Each point that you use will decrease the amount of points of the other person by three. For instance, if you use 5 points you will remove 15 points from the other person.

The other person will also have to decide how many points wants to use to decrease your amount of points according to what you contributed in the last round.

At the end of the experiment, the system will randomly implement one of your decisions or one of those of the other participant. If you don't take a decision in this phase, you want gain anything from this part of the experiment.

How many points you want to use to decrease other person's amount if that person:

- invested less than 50 points in the 28th round?
- invested 50 points in the 28th round?
- invested more than 50 points in the 28th round?

After punishment decision

Now we ask you to think how many points the other person used in the previous question. Specifically, we ask you how much do you think the other persons in the experiment used to decrease the gains of their paired participant. Remember that all of you received 30 points and have been asked to use some of them to reduce the gains of their paired participant as a function of what they did in the 28th round.

Each of your answers will be compared with the average answers from all participants in this experiment and if you will estimate this quantity correctly enough you will gain 10 additional points. You can gain these points for each correct answer. The closer your answer is to the average the more you gain. If you don't take a decision in this phase, you want gain anything from this part of the experiment.

How many points do you think that the other participants in this experiment used to decrease the gain of their paired participant if:

- the other paired participant invested less than 50 points in the 28th round?
- the other paired participant invested 50 points in the 28th round?
- the other paired participant invested more than 50 points in the 28th round?

1.2 Social norm belief elicitation method

During our experiment, we collect a set of expectations to assess the presence of a social norm, its endorsement and its causal effect on actual behavior.

According to Bicchieri,[1] people's minds are characterized by a web of beliefs that motivates their behaviors. Some of those beliefs are personal and defined by people's attitudes or creeds, some others are called social expectations because they are based on what others do. Within the first set of beliefs the Personal Normative Beliefs are those about which actions the person thinks should be done in specific circumstance. We associate such beliefs to the so called first-order beliefs. On the other hand, social beliefs are divided in two categories. We have empirical expectations that express people's expectations concerning what *others* do in specific circumstance, and normative expectations that are beliefs about what *others* think should be done. The normative expectations are second order beliefs- beliefs about what others believe, that is, "beliefs about beliefs" and sometimes they are also accompanied by a certain willingness to punish deviants.

A social norm is a collective practice sustained by empirical and normative expectations and by preferences conditional on both these expectations. Bicchieri's definition, hence, implies that the two expectations need to be mutually active and elicited when assessing the presence of a social norm. If empirical and normative expectations do not mutually inform the behavior of people, then no social norm is present but other norms could be in place (i.e. descriptive norms, customs, morals).

The elicitation methods applied in the manuscript considers such requirement. Moreover, in order to ensure truth-telling the elicitation questions are incentivized such that participants received ten additional points for each exact answer (and less points the more their answers were inaccurate). We elicited expectations in an ordered way *and* we incentivised them based on this ordering. That is, subjects knew that we would compare their ordered list of responses (five items) to the ordered list of true values for their group (empirical expectations to contributions and personal normative beliefs to normative expectations) and we would compare the two to calculate how much they earned. The closer they matched, the more they earned.

However, the order in which the norm-elicitation and behavioral experiments are conducted may systematically affect the elicited norms and behavior. On the one hand, eliciting norms after having elicited behavior may introduce systematic biases in the measurement of norms like self-serving biases.[5, 7] On the other, if elicitation occurs before the behavior, people may focus their attention on the norms that prevail in that situation, and may thus affect behaviour focus their attention on the norms that prevail in that situation, and may thus affect behaviour, and in doing so they incur in what is called situational cues biases.[2, 3, 4, 10] To avoid those biases, we elicited people's beliefs sometimes before and sometimes after their behavioral choice with the order of beliefs elicitation was randomly selected.

The presence of social norm and its endorsement are not the same phenomenon. In fact, the endorsement of a social norm implies that people, following the norm, are willing to punish the deviants. Hence, people's willingness to punish needs to be elicited along with the expectations of others' willingness to punish. Yet, given that the paper aims to study the emergence and presence of a social norm, rather than its endorsement, the elicitation of both people's punishment and people's expectations on others' punishment has been done only at the last round of the game (30th day).

Finally, the emergence and presence of a social norm might affect people's in-game behavior. Yet, to study the causal connection between the presence of a social norm and people behavior we need to measure how much people are willing to change their behavior if that social norm were to change. To do so, we created a conditional contribution scenario in which people were

asked to contribute conditional on if the majority of their group members put in [*at least 50 points/less than 50 points*] and believe that you should all spend [*at least 50 points/less than 50 points*]. We incentivized this by identifying the EE and NE combination that held in the subjects' group and they were additionally paid for this. This elicitation took place in rounds 1, 5, 10, 14, 15, 19, 24, and 28 and was used for the causal analysis and the base for building the analysis of behavioral typology.

1.3 Social norms strength definition and measurement

All three measures (consistency, accuracy, and specificity) are necessary for a social norm to exist. If expectations are not consistent nor accurate, then, respectively, there is no coordination in expectations and expectations do not reflect reality. In both cases coordinated behavior is precluded. While if a norm is unrestricted in terms of the socially approved action, then it is meaningless. Our three measures are calculated considering the ordered ranking of all the set of individual's expectations within the groups and across treatment. For each participant, p, in group G_p that contains other m individuals, playing at time t we rank, (R is the rank order), participant's expectations and contributions. Further, we define G_p^{-p} as the group G_p excluding participant p, that contains $(m-1)$ individuals. For example, $EE_R^p(t)$ is the ranked list of all the EE of participant p playing at time t. The ranked ordered allow us to have a granular vision of individuals' expectations.

Consider the following example, which uses the measure of specificity, to illustrate why it is essential to use distributions instead of only averages. If we were to use each individual's average responses the measure would conflate situations in which people believe there are two (or more) norms within a group and those that believe there is a single norm. Imagine that a group's average empirical expectation is 50. Individual i in that group reports her empirical expectations as $(0, 0, 50, 100, 100)$ while individual j, also in that group, thinks that the distribution is $(50, 50, 50, 50, 50)$. If we use the averaging approach, then both individuals receive a score of 0 and are thus interchangeable (since the group average is 50 and the average expectation for each is also 50). Conversely, with our approach i receives a score of 200 $(|0-50| + |0-50| + |50-50| + |100-50| + |100-50|)$ while j receives a score of 0 $(|50-50| + |50-50| + |50-50| + |50-50| + |50-50|)$ allowing us to separate between the radically different situations.

1.3.1 Consistency

Consistency may be defined as the difference among individual's expectations and group's expectations that are both ordered by the rank (Eq. (1)) $EE_R^p(t)$ and $NE_R^p(t)$ are the ordered empirical and normative expectations of participant p in group g at time t. Since individuals do not know the identity of the other people in their group, we can order their expectations and compare their orderings with the others in their group. In fact, $EE_R^p(t)$ and $NE_R^p(t)$ are the ordered empirical and normative expectations of individuals belonging to the group G_p^{-p} at time t. We calculate consistency of the entire population at time t in the following way:

$$Consistency(t) = 1 - \frac{\Sigma_p \Sigma_{q_p^{-p}} \Sigma_{R\in[1,m-1]}(|EE_R^p(t) - EE_R^q(t)| + |NE_R^p(t) - NE_R^q(t)|)}{max\{\Sigma_p \Sigma_{q_p^{-p}} \Sigma_{R\in[1,m-1]}(|EE_R^p(t) - EE_R^q(t)| + |NE_R^p(t) - NE_R^q(t)|)\}} \tag{1}$$

As the deviation between participant p's ranked EE (NE) and his group mates' ranked EE (NE). For example, let us imagine that participant p has the following ordered EE $(99, 80, 70, 40, 0)$, that is he expects that at least one of his group mates will contribute 99, another 80, another 70 and so forth, he, of course, does not know who will contribute 99 or 80 or 70 etc. Now imagine that one of his group mates has the following ordered EE list $(90, 72, 50, 35, 20)$. We calculate the deviance between the two lists for each rank, for example for the first rank the deviance will be of 9 points $(|90-99| = 9)$, and so forth with the rest of the ranks and participants.

Consistency measures how far participants' expectations, about how many other participants are like-minded, i.e. they are different from one to another. The smaller the EE and NE deviance

(i.e. the deviance is closed to 0) among participants, the higher is the match of expectations within each group. When consistency is high, i.e. near to 1, the distributions of expectations within each group match. When consistency is low, hence equal to 0, the deviances among expectations is maximal and there is no common knowledge within the group about how the others will and should behave. All participants have different expectations. When consistency reach a level of nearly 0,5 we are presented with a situation in which several different beliefs coexist.

1.3.2 Accuracy

Through accuracy we measure whether the ranks of ordered empirical and normative expectations of an individual are close (hence accurate), respectively, to the ranks of ordered cooperation level of the other participants of the group (i.e. $C_R^{G_p^{-p}}(t)$) and the ranks of ordered personal normative beliefs of the participants in the group (i.e. $PNB_R^{G_p^{-p}}(t)$) (Eq. (2)). We calculate accuracy of the entire population at time t in the following way:

$$Accuracy(t) = 1 - \frac{\Sigma_p \Sigma_{R\in[1,m-1]}(|EE_R^p(t) - C_R^{G_p^{-p}}(t)| + |NE_R^p(t) - PNB_R^{G_p^{-p}}(t)|)}{max\{\Sigma_p \Sigma_{R\in[1,m-1]}(|EE_R^p(t) - C_R^{G_p^{-p}}| + |NE_R^p(t) - PNB_R^{G_p^{-p}}(t)|)\}} \quad (2)$$

For example, let us consider participant p's ordered EE $(99, 80, 70, 40, 0)$. Imagine that from his group mates we have their cooperation levels, for example$(45, 0, 78, 23, 85)$ and hence we can create an ordered list of such levels, i.e. $(85, 78, 45, 23, 0)$ and compare it with the ordered EE of participant p. We calculate the deviance between the two, for example the difference between the highest EE and the highest contribution is of 14 point $(= |99 - 85|)$, the second highest difference is of 2 points, the third difference is of 15 points and so forth: meaning that participant p overestimates the number of people that she expects will contribute. The same overshooting could happen when we compare participant's NE and the others' PNB.

Accuracy is a measure of how much participants' expectations (both empirical and normative) "forecast" what the others will do or think that is appropriate to do. Or in other words, how much the expectations are far (i.e. overshooting) or close (i.e. accuracy) from what the others will do and from what they believe is the right thing to do.

When accuracy is high, then there is no difference between what participants expect from the others' contributions and their actual contributions and there is no difference between participants' normative expectations and what the others believe is appropriate to do. A convergence towards a unique strategy on how to resolve the dilemma occurs in a faster time. When accuracy starts decreasing (i.e. less than 1), such differences, on both empirical and normative perspective, is wide hence the common strategy will need larger time to emerge. From this measure we are not able to identify the nature of the common strategy (i.e. cooperation or defection), we only know that participants will coordinate on a common strategy.

1.3.3 Specificity

Through specificity we measure the extent to which situations differ in the range of behavioral responses that are considered appropriate, or the extent to which the situation constrains or affords opportunities for behavioral options (Eq. (3)). This construct comes from the theoretical and empirical work.[6, 8] We measure whether the individual's empirical and normative expectations are close, respectively, to the group average empirical and normative expectations.

Roughly put, for individual p in group G_p, and the average of each measure is calculated at the group level. We calculate specificity of the entire population at time t in the following way:

$$Specificity(t) = 1 - \frac{\Sigma_p \Sigma_{R\in[1,m-1]}(|EE_R^p(t) - \overline{EE_R^{G_p}(t)}| + |NE_R^p(t) - \overline{NE_R^{G_p}(t)}|)}{max\{\Sigma_p \Sigma_{R\in[1,m-1]}(|EE_R^p(t) - \overline{EE_R^{G_p}(t)}| + |NE_R^p(t) - \overline{NE_R^{G_p}(t)}|)\}} \quad (3)$$

Specificity measures how much participant's expectations are far from the "average mind" of the group.

We can finally calculate norm strength in the following way:

$$norm\ strength = consistency \times accuracy \times specificity \quad (4)$$

This way of calculating norm strength implies that all components are necessary for a norm to exists. To help with the interpretation of norm strength, we created the Random Decision Baseline. This reflects what values for social norm strength components would be observed if subjects were making completely random decisions, i.e. uniformly distributed between 0 and 100, for contribution, empirical expectations, normative expectations, and personal normative belief. This scenario represents the baseline case for the values of consistency (0.6895), accuracy (0.8133), specificity (0.7552) and social norm strength (0.4235) for all rounds and both treatments. The computed values are the average of 1000 simulated setups.

1.4 Analysis of behavioral typology

The four conditional contributions can be summarized into two measures of responsiveness to empirical and normative expectations: Empirical Expectation Influence (EEI), which represents subjects' sensitivity to empirical expectations (Eq. (5)), and Normative Expectation Influence (NEI), which represents subjects' sensitivity to normative expectations (Eq. (6)). We consider individual conditional contributions C^i_{xy} as the contribution of subject i, averaged across all subject's answers to conditional contributions, in the scenario in which empirical expectations are set to be x (h: high, l: low) and normative expectations to be y (h: high, l: low). The measure EEI (NEI) quantifies the change in contribution from a scenario in which empirical (normative) expectations are low to a scenario in which empirical (normative) expectations are high, while keeping normative (empirical) expectations fixed. Specifically, we have that:

$$EEI^i = \frac{(C^i_{hh} - C^i_{lh}) + (C^i_{hl} - C^i_{ll})}{2} \tag{5}$$

and

$$NEI^i = \frac{(C^i_{hh} - C^i_{lh}) + (C^i_{hl} - C^i_{ll})}{2} \tag{6}$$

Like in Wave 1, through these responsiveness measures we are able to identify five clusters of behaviors. The numerosity of the clusters is chosen by the k-means clustering algorithm, that groups the data in a way that it both minimizes the dispersion within clusters and maximizes the distance among centroids of different clusters. The algorithm, that does not assume beforehand any specific number of types of behaviors, finds that $k = 5$ clusters is the optimal number of groups according to the Davies-Bouldin index in both waves. Moreover, the number of types is also supported by theoretical assumptions in related works where similar types are defined according to qualitative procedures.

2 Effect of Covid-19 hypotheses

Table S1: Summary of hypotheses and results for the replication study (W2).

	Hypotheses	All	HL90	HL60	LH60	LH90
1	Covid-19 increases average contributions compared to W1	0	0	0	0	0
2	Covid-19 increases the likelihood of reaching the threshold compared to W1	0	0	−	+	+
3	Covid-19 increases the average EE compared to W1	0	0	−	+	0
4	Covid-19 increases the average NE compared to W1	0	0	−	0	0
5	Covid-19 increases norm strength compared to W1	+	0	−	+	+
6a	Increasing manipulated NE increases contributions	+				
7b	Increasing manipulated EE increases contributions for some and decreases for others	+				

Note: The column "All" shows the results of the preregistered hypotheses. The columns LH60 and HL60 show the results for the Low High treatment and the High Low treatment under 60% risk, respectively.

Hypotheses 1–5 test the effect of the Covid-19 pandemic. Hypothesis 6 tests the exploratory finding from Wave 1 that the effect of empirical expectations is more heterogeneous (resulting negative for a subgroup of subjects) than that of normative expectations.

2.1 H1: Covid-19 increases the average contribution

2.1.1 Analytical strategy

To test whether contributions increased during the Covid-19 pandemic we tested the difference in contributions for Wave 1 and Wave 2. We used multilevel Ordinary Least Squares regressions. Model 1 tests the main effect of Wave 2, Model 2 includes controls for the risk level (High collective risk), the treatment (Low to High), empirical expectations, normative expectations, and personal normative beliefs. Model 3 includes controls for personal preferences and psychological variables (Social Value Orientation, risk preferences, Autism Spectrum Quotient, and Big Five) and Model 4 includes sociodemographic controls. (age, gender, whether respondent is a student, in how many experiments they participated, and their political orientation). Finally, Model 5 includes the interactions between Wave, risk level, and treatment (i.e., three two-way and one three-way interaction).

From Model 5, the effect of the wave difference for each treatment $\times$ risk level combination can be derived as follows:

- Effect(Wave 2 | Low to High, Low collective risk) = Wave 2 + Wave 2 $\times$ Low to High
- Effect(Wave 2 | Low to High, High collective risk) = Wave 2 + Wave 2 $\times$ High collective risk + Wave 2 $\times$ Low to High + Wave 2 $\times$ High collective risk $\times$ Low to High
- Effect(Wave 2 | High to Low, High collective risk) = Wave 2 + Wave 2 $\times$ High collective risk
- Effect(Wave 2 | High to Low, Low collective risk) = Wave 2

To facilitate the interpretation of the interactions, we calculate the marginal effect for each combination. Marginal effects measure the impact that an instantaneous unit change in one variable has on the outcome variable while all other variables are held constant. In Stata, we use the `margins` command to calculate the overall marginal effect of Wave 1 and Wave 2 (based on Model 4) as well as for each treatment $\times$ risk level combination (based on Model 5). We then calculate whether the marginal effects for Wave 1 and Wave 2 are significantly different from one another using the `lincom` command. The result of these difference tests correspond to the effects described above. The marginal effects for each category, as well as the result of the difference test, are reported in Table 2 in the main paper.

2.1.2 Results

Covid-19 did not result in an overall increase in contribution rates in Wave 2 (50.36) compared to Wave 1 (49.59) (see Table S2; $b = 0.768$, $p = 0.171$, 95% CIs $[-0.33, 1.87]$), rejecting hypothesis 1. Even if we zoom in on treatment and risk level effects, there are no significant differences between Wave 1 and Wave 2 for any of the treatment $\times$ risk level combination (see Figure S1).

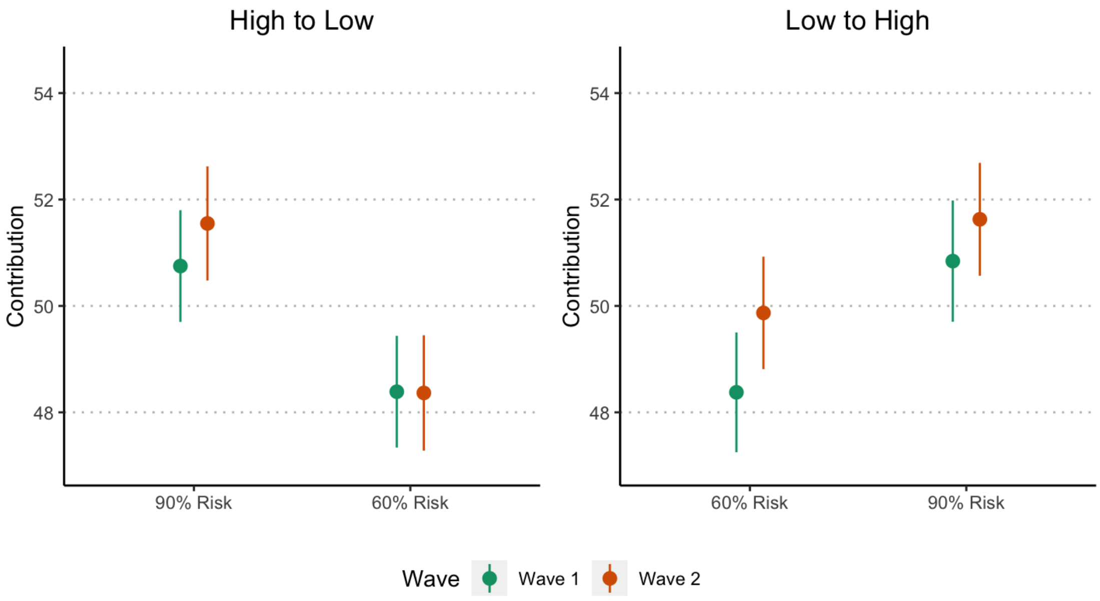

Figure S1: Average Contribution by Wave, Treatment, and Risk Level

Table S2: Average Contribution by Wave, Treatment, and Risk Level

	Model 1	Model 2	Model 3	Model 4	Model 5
Wave 2	−0.237	0.075	0.337	0.768	−0.023
	(0.698)	(0.521)	(0.505)	(0.560)	(0.775)
High collective risk		2.417***	2.416***	2.417***	2.362***
		(0.158)	(0.158)	(0.158)	(0.300)
Low (0.6) to High (0.9)		0.479	0.370	0.415	−0.011
		(0.521)	(0.504)	(0.503)	(0.750)
Wave 2 × High coll. risk					0.823
					(0.428)
Wave 2 × Low (0.6) to High (0.9)					1.515
					(1.052)
High coll. risk × Low (0.6) to High (0.9)					0.104
					(0.444)
Wave 2 × High coll. risk × Low (0.6) to High (0.9)					−1.530*
					(0.618)
Empirical expectations		0.446***	0.447***	0.447***	0.444***
		(0.017)	(0.017)	(0.017)	(0.017)
Normative expectations		0.187***	0.186***	0.186***	0.183***
		(0.019)	(0.019)	(0.019)	(0.019)
Personal normative beliefs		0.448***	0.449***	0.448***	0.445***
		(0.012)	(0.012)	(0.012)	(0.012)
Social Value Orientation angle			0.091***	0.087***	0.087***
			(0.021)	(0.022)	(0.022)
Risk preferences			−0.666***	−0.592***	−0.587***
			(0.170)	(0.171)	(0.171)
Autism Spectrum Quotient			−0.052	−0.034	−0.039
			(0.051)	(0.051)	(0.052)
Big Five					
Extraversion			−0.065	−0.070	−0.072
			(0.046)	(0.046)	(0.046)
Agreeableness			0.034	0.018	0.017
			(0.058)	(0.058)	(0.058)
Conscientiousness			−0.001	−0.028	−0.028
			(0.045)	(0.046)	(0.046)
Neuroticism			0.019	−0.018	−0.020
			(0.040)	(0.043)	(0.043)
Openness			0.077	0.085	0.086*
			(0.044)	(0.044)	(0.044)
Age				0.005	0.004
				(0.031)	(0.031)
Gender					
Female				1.437*	1.455**
				(0.562)	(0.563)
Other				0.379	0.402
				(3.021)	(3.024)
Student				−0.529	−0.560
				(0.701)	(0.702)
Experienced				−0.181	−0.181
				(0.161)	(0.161)
Political orientation (1-7)				0.056	0.053
				(0.171)	(0.171)
Constant	50.231***	−7.328***	−10.200**	−9.171*	−8.251*
	(0.498)	(0.974)	(3.601)	(3.780)	(3.821)
Random intercept	2.089***	1.781***	1.739***	1.729***	1.730***
	(0.032)	(0.033)	(0.033)	(0.033)	(0.033)
Residual variance	2.390***	2.244***	2.244***	2.244***	2.244***
	(0.006)	(0.006)	(0.006)	(0.006)	(0.006)
Observations	15195	15181	15181	15181	15181

Note: SEs in parentheses. * $p < 0.05$, ** $p < 0.01$, *** $p < 0.001$

2.2 H2: Covid-19 increases the likelihood of groups reaching the threshold

2.2.1 Analytical strategy

To test whether Covid-19 increased cooperation we move the analysis to the group level and test whether groups are more likely to reach the threshold of 300 points using a multilevel logistic regression. Since the analyses are on the group level there are no control variables. Model 1 tests the main effect for the wave differences, Model 2 adds the controls for treatment and high collective risk, and Model 3 includes the interactions. As for the earlier analyses (see section 2.1.1), we calculated the marginal effects. Since we ran a logistic regression, the marginal effects represent the probability that the threshold is reached in each wave and for each treatment × risk level condition.

2.2.2 Results

There is no difference in the overall likelihood of groups reaching the threshold between Wave 1 (0.62) and Wave 2 (0.63) ($b = 0.062$, $p = 0.469$, 95% CIs $[-0.11, 0.22]$), rejecting Hypothesis 2 (see Table S3). However, the overall effect masks differential effects for the two treatments. In the High Low treatment, there is no difference in the first part (90% risk), but the likelihood of reaching the threshold substantially dropped when risk decreased to 60% (0.47 vs 0.31, $b = -0.160$, $p < 0.001$, 95% CIs $[-0.23, -0.09]$). In the Low High treatment, on the other hand, groups in Wave 2 were significantly better in coordinating when risk was low (0.49 vs 0.62, $b = 0.129$, $p = 0.001$, 95% CIs $[0.05, 0.21]$) and continued to coordinate better when risk increased to 90% (0.67 vs 0.81, $b = 0.139$, $p < 0.001$, 95% CIs $[0.07, 0.21]$).

Table S3: Likelihood of reaching the threshold by Wave, Treatment, and Risk Level

	Model 1	Model 2	Model 3
Wave 2	0.077	0.062	-0.682^{***}
	(0.081)	(0.086)	(0.162)
Low (0.6) to High (0.9)		0.283^{***}	0.090
		(0.086)	(0.159)
High collective risk		1.360^{***}	1.620^{***}
		(0.087)	(0.176)
Wave 2 × Low (0.6) to High (0.9)			1.208^{***}
			(0.229)
Wave 2 × High collective risk			0.455
			(0.251)
Low (0.6) to High (0.9) × High collective risk			-0.854^{***}
			(0.246)
Wave 2 × Low (0.6) to High (0.9) × High collective risk			-0.235
			(0.354)
Constant	0.464^{***}	-0.282^{***}	-0.130
	(0.058)	(0.081)	(0.109)
Observations	2588	2588	2588

Note: SEs in parentheses. * $p < 0.05$, ** $p < 0.01$, *** $p < 0.001$

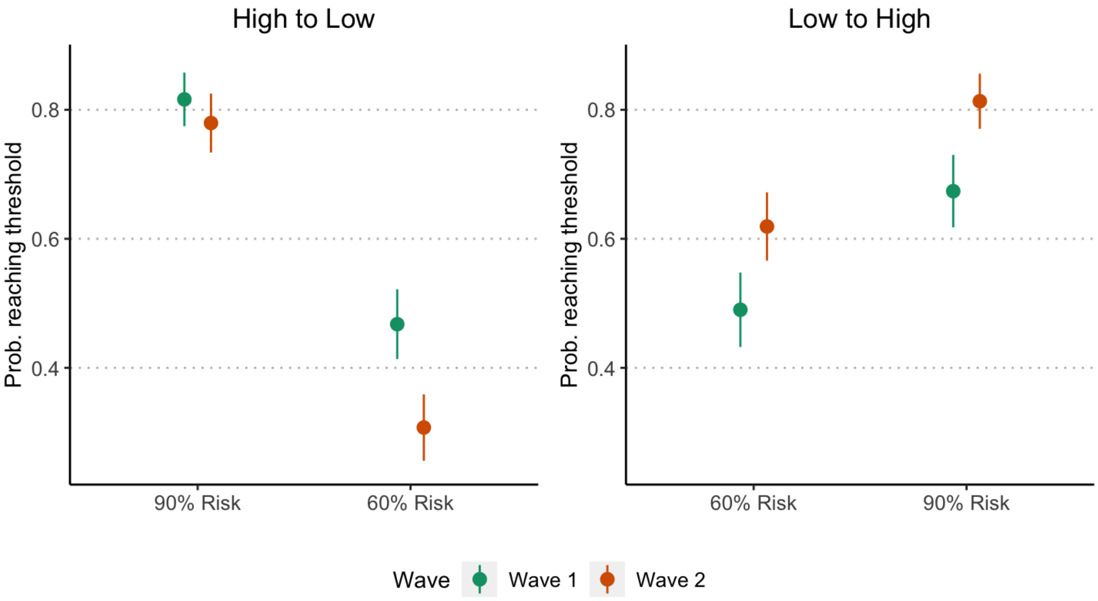

Figure S2: Probability of reaching the threshold by Wave, Treatment, and Risk Level

2.3 H3 & 4: Covid-19 increases EE and NE

2.3.1 Analytical strategy

To test whether contributions increased during the Covid-19 pandemic we followed an analytical strategy similar to that of the wave difference test with respect to contributions (see section 2.1.1). The only difference is that the regressions do not control for empirical expectations, normative expectations, and personal normative beliefs.

2.3.2 Results

Neither for Empirical Expectations (51.20 in W1 vs 50.84 in W2) nor for Normative Expectations (52.02 in W1 vs 51.80 in W2) did Covid-19 result in an overall average increase, rejecting Hypotheses 3 and 4 (see Tables S4 and S5; EE: $b = 0.352$, $p = 0.301$; 95% CIs $[-1.02, 0.31]$; NE: $b = -0.220$, $p = 0.532$, 95% CIs $[-0.91, 0.47]$). Zooming in on differences between treatments and risk levels, Covid-19—as measured by the comparison between Wave 1 and Wave 2—did result in some changes (see Model 5 in Tables S4 and S5 and Figure S3).

When the risk level is 90%, regardless of the treatment, there are no differences between Wave 1 and Wave 2 (EE High Low 53.20 vs 52.81 in W1 and W2, $b = -0.391$, $p = 0.393$, 95% CIs $[-1.29, 0.51]$; EE Low High 51.61 vs 51.66 in W1 and W2, $b = 0.055$, $p = 0.907$, 95% CIs $[-0.88, 0.99]$; NE High Low 53.41 vs 53.17 in W1 and W2, $b = -0.242$, $p = 0.611$, 95% CIs $[-1.17, 0.69]$; NE Low High 51.71 vs 51.73 in W1 and W2, $b = 0.021$, $p = 0.966$, 95% CIs $[-0.94, 0.99]$). Expectations were above 50 in Wave 1 (signalling cooperative attitudes and behavior) and do not increase further in Wave 2.

For a risk level of 60%, Wave 2 did result in an increase in empirical expectations in the Low High treatment, bringing the expectations above 50 (49.60 vs 50.95, $b = 1.349$, $p = 0.004$, 95% CIs $[0.43, 2.27]$). The normative expectations for Low High, already above 50 in Wave 1, did not change significantly (51.60 vs 52.39, $b = 0.786$, $p = 0.108$, 95% CIs $[-0.17, 1.75]$). For High Low, when risk is 60%, the opposite occurs. Both empirical expectations and normative expectations decrease significantly compared to Wave 1 (EE 50.20 vs 47.79, $b = -2.41$, $p < 0.001$, 95% CIs $[-3.31, -1.51]$; NE 51.18 vs 49.74, $b = -1.44$, $p = 0.003$, 95% CIs $[-2.38, -0.51]$).

Table S4: Average Empirical Expectations by Wave, Treatment, and Risk Level

	Model 1	Model 2	Model 3	Model 4	Model 5
Wave 2	−0.496	−0.555	−0.595	−0.352	−2.410***
	(0.307)	(0.306)	(0.304)	(0.340)	(0.459)
High collective risk (0.9)		2.694***	2.695***	2.695***	3.003***
		(0.090)	(0.090)	(0.090)	(0.172)
Low (0.6) to High (0.9)		0.053	−0.007	−0.032	−0.595
		(0.306)	(0.304)	(0.305)	(0.445)
Wave 2 × High coll. risk					2.019***
					(0.248)
Wave 2 × Low to High					3.759***
					(0.624)
High coll. risk × Low to High					−0.999***
					(0.257)
Wave 2 × High coll. risk × Low to High					−3.312***
					(0.359)
Social Value Orientation angle			0.025*	0.023	0.022
			(0.013)	(0.013)	(0.013)
Risk preferences			−0.048	−0.049	−0.034
			(0.102)	(0.103)	(0.102)
Autism Spectrum Quotient			−0.016	−0.009	−0.021
			(0.031)	(0.031)	(0.031)
Big Five					
Extraversion			0.053	0.057*	0.050
			(0.028)	(0.028)	(0.028)
Agreeableness			−0.029	−0.028	−0.030
			(0.035)	(0.035)	(0.035)
Conscientiousness			0.040	0.033	0.035
			(0.027)	(0.028)	(0.028)
Neuroticism			−0.011	−0.010	−0.014
			(0.024)	(0.026)	(0.026)
Openness			−0.043	−0.041	−0.040
			(0.026)	(0.026)	(0.026)
Age				0.005	0.003
				(0.019)	(0.019)
Gender					
Female				0.001	0.046
				(0.341)	(0.335)
Other				−1.120	−0.992
				(1.832)	(1.799)
Student				−0.339	−0.408
				(0.425)	(0.417)
Experienced				−0.092	−0.088
				(0.098)	(0.096)
Political orientation (1-7)				0.028	0.019
				(0.104)	(0.102)
Constant	51.259***	49.931***	49.889***	49.793***	50.718***
	(0.219)	(0.265)	(2.108)	(2.231)	(2.211)
Random intercept	1.251***	1.249***	1.235***	1.232***	1.213***
	(0.033)	(0.033)	(0.033)	(0.033)	(0.033)
Residual variance	1.742***	1.712***	1.712***	1.712***	1.702***
	(0.006)	(0.006)	(0.006)	(0.006)	(0.006)
Observations	15188	15188	15188	15188	15188

Note: SEs in parentheses. * $p < 0.05$, ** $p < 0.01$, *** $p < 0.001$

Table S5: Average Normative Expectations by Wave, Treatment, and Risk Level

	Model 1	Model 2	Model 3	Model 4	Model 5
Wave 2	−0.419	−0.452	−0.458	−0.220	−1.441**
	(0.318)	(0.318)	(0.317)	(0.352)	(0.477)
High collective risk		1.315***	1.316***	1.316***	2.231***
		(0.084)	(0.084)	(0.084)	(0.159)
Low (0.6) to High (0.9)		0.081	0.041	−0.001	0.422
		(0.318)	(0.316)	(0.316)	(0.462)
Wave 2 × High coll. risk					1.200***
					(0.229)
Wave 2 × Low (0.6) to High (0.9)					2.228***
					(0.648)
High coll. risk × Low (0.6) to High (0.9)					−2.120***
					(0.237)
Wave 2 × High coll. risk × Low (0.6) to High (0.9)					−1.965***
					(0.332)
Social Value Orientation angle			0.021	0.021	0.020
			(0.013)	(0.014)	(0.013)
Risk preferences			−0.086	−0.085	−0.077
			(0.106)	(0.107)	(0.107)
Autism Spectrum Quotient			−0.035	−0.029	−0.036
			(0.032)	(0.032)	(0.032)
Big Five					
Extraversion			0.023	0.026	0.021
			(0.029)	(0.029)	(0.029)
Agreeableness			−0.039	−0.034	−0.036
			(0.036)	(0.037)	(0.036)
Conscientiousness			0.054	0.043	0.045
			(0.029)	(0.029)	(0.029)
Neuroticism			0.013	0.012	0.010
			(0.025)	(0.027)	(0.027)
Openness			−0.002	0.005	0.005
			(0.027)	(0.027)	(0.027)
Age				−0.023	−0.024
				(0.020)	(0.020)
Gender					
Female				0.147	0.179
				(0.353)	(0.351)
Other				−1.402	−1.297
				(1.901)	(1.885)
Student				−0.721	−0.765
				(0.441)	(0.437)
Experienced				−0.113	−0.108
				(0.101)	(0.100)
Political orientation (1-7)				0.150	0.146
				(0.108)	(0.107)
Constant	52.111***	51.438***	50.472***	50.832***	50.994***
	(0.227)	(0.275)	(2.194)	(2.315)	(2.316)
Random intercept	1.299***	1.297***	1.286***	1.279***	1.271***
	(0.033)	(0.033)	(0.033)	(0.033)	(0.033)
Residual variance	1.644***	1.636***	1.636***	1.636***	1.623***
	(0.006)	(0.006)	(0.006)	(0.006)	(0.006)
Observations	15183	15183	15183	15183	15183

Note: SEs in parentheses. * $p < 0.05$, ** $p < 0.01$, *** $p < 0.001$

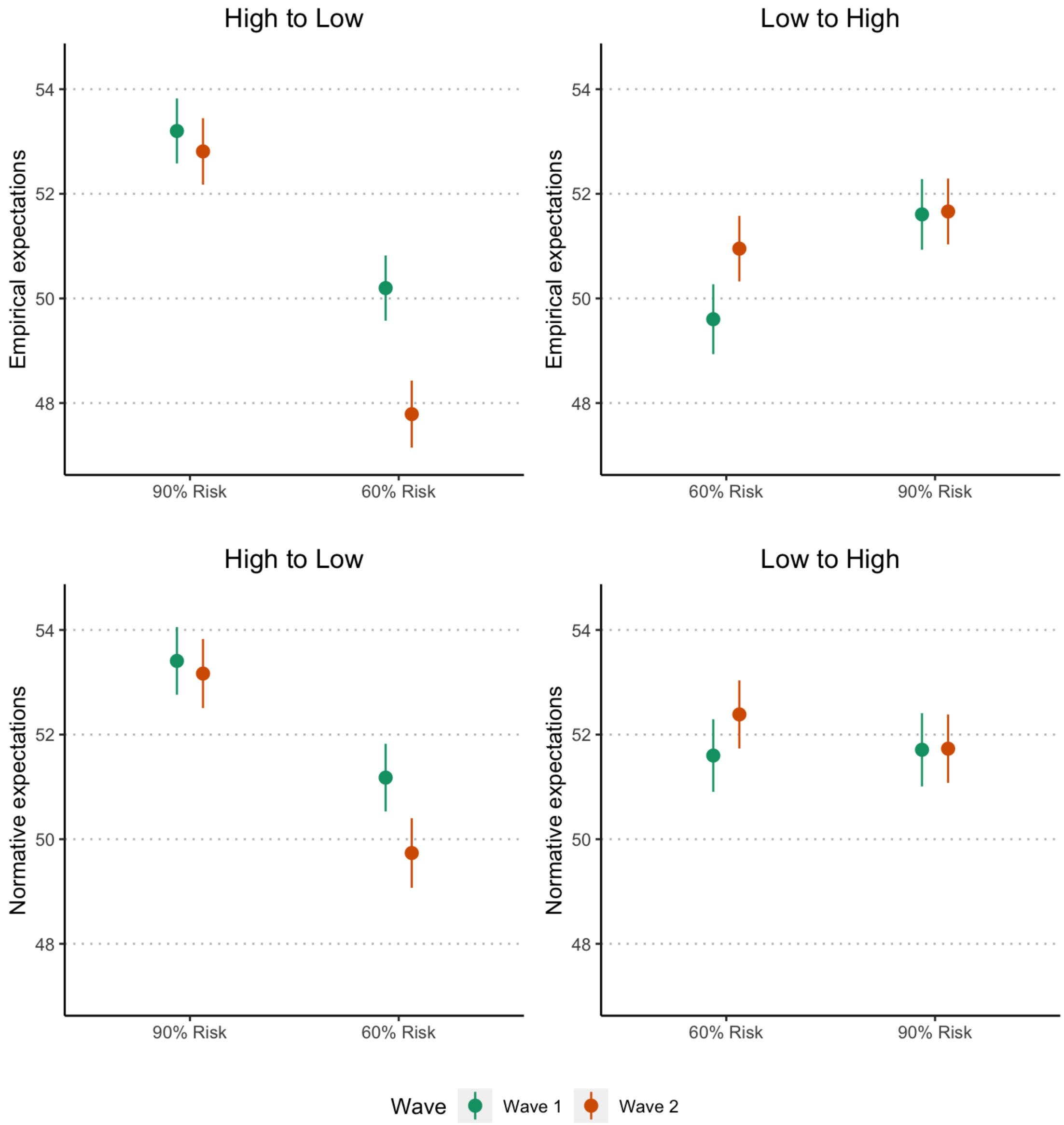

Figure S3: Average Empirical and Normative Expectations by Wave, Treatment, and Risk Level

2.4 H5: Covid-19 increases norm strength

2.4.1 Analytical strategy

To evaluate the strength of norms, in [9] an index of strength was developed based on three criteria: (i) agreement between expectations of group members (consistency), (ii) whether expectations correctly predict the behavior and personal normative beliefs of others (accuracy), and (iii) how specific the norm is concerning the range of acceptable behaviors (specificity). (see Supplementary Information Section 1.3 for precise definitions and the measurement scale). We used the same index to test the hypothesis that Covid-19 increased norm strength (H4).

In Table S6 we test the strength of social norms using a multilevel linear regression. Each observation represents the social norm strength of one group in a round. We exclude groups in which three or more participants were inactive for any of the relevant responses (contributions or expectations). Since the analyses are on the group level, the build up in Models 1–3 is the same as in section 2.2.1. As exploratory analyses, we repeat the analyses for consistency, accuracy, and specificity separately to test whether norm strength improved on all indicators (see Tables S7–S9).

2.4.2 Results

There is indeed an overall positive effect of Wave 2 on norm strength, supporting the hypothesis that Covid-19 increased norm strength compared to Wave 1 ($b = 0.015$, $p = 0.001$, 95% CIs $[0.01, 0.02]$, Table S6). Social expectations in Wave 2 are more accurate overall ($b = 0.005$, $p = 0.001$, 95% CIs $[0.002, 0.008]$, see Table S7), more consistent across group members ($b = 0.007$, $p = 0.004$, 95% CIs $[0.002, 0.012]$, see Table S8), and more specific in the range of acceptable behavior ($b = 0.005$, $p = 0.020$, 95% CIs $[0.001, 0.009]$, see Table S9).

If we break down the results by treatment and risk level, we find that this effect is driven by the overall increase in norm strength in the Low High treatment (see Model 3 in Table S6 and Figure S4). For this treatment, norm strength increased from 0.68 to 0.73 when risk was 60% ($b = 0.054$, $p < 0.001$, 95% CIs $[0.04, 0.07]$) and from 0.81 to 0.85 when risk increased to 90% ($b = 0.041$, $p < 0.001$, 95% CIs $[0.02, 0.06]$). The increase in norm strength is the result of increased accuracy, consistency, and specificity both when risk was 60% and when risk was 90% (Tables S7, S8, and S9).

In the High Low treatment, on the other hand, social norms are equally strong across waves if risk is 90% ($b = 0.005$, $p = 0.521$, 95% CIs $[-0.01, 0.02]$). All three components of norm strength are similar across waves. When risk decreases to 60%, norm strength decreases from 0.75 in Wave 1 to 0.71 Wave 2 ($b = -0.038$, $p < 0.001$, 95% CIs $[-0.05, -0.02]$). While the accuracy in social expectations is similar across waves ($b = 0.005$, $p = 0.085$, 95% CIs $[-0.011, 0.001]$), the consistency across group members decreases ($b = -0.021$, $p < 0.001$, 95% CIs $[-0.030, -0.012]$), and the range range of acceptable behavior becomes wider (i.e., less specificity, $b = -0.022$, $p < 0.001$, 95% CIs $[-0.029, -0.014]$).

Table S6: Social Norm Strength by Wave, Treatment, and Risk Level

	Model 1	Model 2	Model 3
Wave 2	0.016***	0.015***	−0.038***
	(0.005)	(0.004)	(0.009)
Low (0.6) to High (0.9)		0.019***	−0.074***
		(0.004)	(0.009)
High collective risk		0.083***	0.017*
		(0.004)	(0.008)
Wave 2 × Low (0.6) to High (0.9)			0.092***
			(0.012)
Wave 2 × High collective risk			0.044***
			(0.012)
Low (0.6) to High (0.9) × High collective risk			0.120***
			(0.012)
Wave 2 × Low (0.6) to High (0.9) × High collective risk			−0.057**
			(0.017)
Constant	0.751***	0.701***	0.750***
	(0.003)	(0.004)	(0.006)
Observations	2588	2588	2588

Note: SEs in parentheses. * $p < 0.05$, ** $p < 0.01$, *** $p < 0.001$

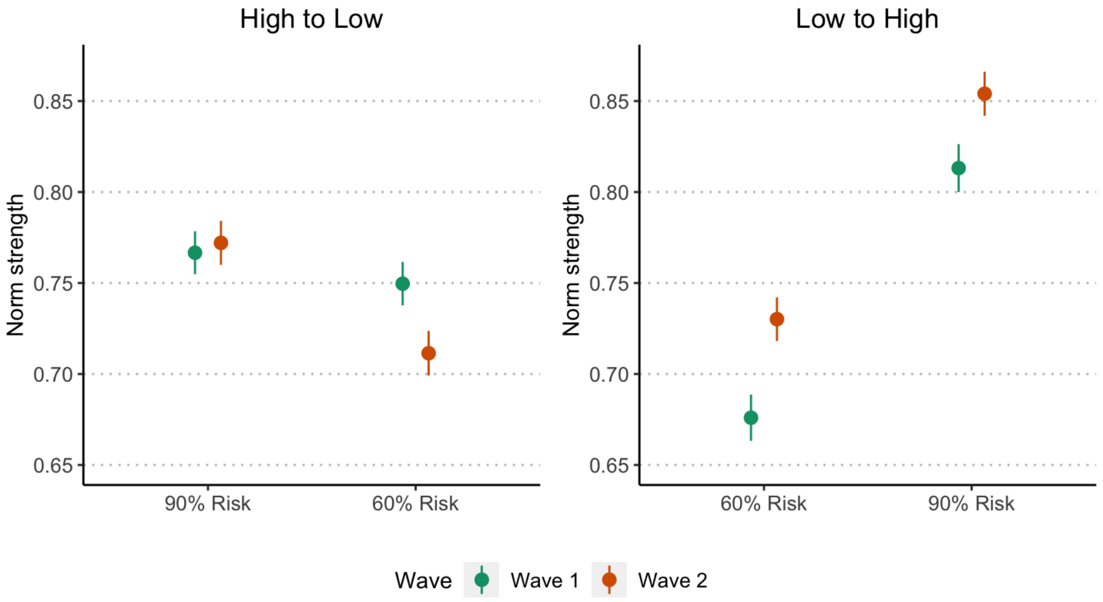

Figure S4: Social Norm Strength by Wave, Treatment, and Risk Level

Table S7: Social Norm Accuracy by Wave, Treatment, and Risk Level

	Model 1	Model 2	Model 3
Wave 2	0.006***	0.005**	−0.005
	(0.002)	(0.002)	(0.003)
Low (0.6) to High (0.9)		0.008***	−0.011***
		(0.002)	(0.003)
High collective risk		0.030***	0.017***
		(0.002)	(0.003)
Wave 2 × Low (0.6) to High (0.9)			0.019***
			(0.004)
Wave 2 × High collective risk			0.008
			(0.004)
Low (0.6) to High (0.9) × High collective risk			0.026***
			(0.004)
Wave 2 × Low (0.6) to High (0.9) × High collective risk			−0.013*
			(0.006)
Constant	0.930***	0.912***	0.922***
	(0.001)	(0.001)	(0.002)
Observations	2644	2644	2644

Note: SEs in parentheses. * $p < 0.05$, ** $p < 0.01$, *** $p < 0.001$

Table S8: Social Norm Consistency by Wave, Treatment, and Risk Level

	Model 1	Model 2	Model 3
Wave 2	0.008**	0.007**	−0.021***
	(0.003)	(0.002)	(0.005)
Low (0.6) to High (0.9)		0.007**	−0.043***
		(0.002)	(0.005)
High collective risk		0.039***	0.002
		(0.002)	(0.005)
Wave 2 × Low (0.6) to High (0.9)			0.047***
			(0.007)
Wave 2 × High collective risk			0.024***
			(0.007)
Low (0.6) to High (0.9) × High collective risk			0.067***
			(0.007)
Wave 2 × Low (0.6) to High (0.9) × High collective risk			−0.028**
			(0.010)
Constant	0.886***	0.863***	0.890***
	(0.002)	(0.002)	(0.003)
Observations	2644	2644	2644

Note: SEs in parentheses. * $p < 0.05$, ** $p < 0.01$, *** $p < 0.001$

Table S9: Social Norm Specificity by Wave, Treatment, and Risk Level

	Model 1	Model 2	Model 3
Wave 2	0.005^{*}	0.005^{*}	-0.022^{***}
	(0.002)	(0.002)	(0.004)
Low (0.6) to High (0.9)		0.006^{**}	-0.035^{***}
		(0.002)	(0.004)
High collective risk		0.033^{***}	0.004
		(0.002)	(0.004)
Wave 2 × Low (0.6) to High (0.9)			0.044^{***}
			(0.005)
Wave 2 × High collective risk			0.023^{***}
			(0.005)
Low (0.6) to High (0.9) × High collective risk			0.051^{***}
			(0.006)
Wave 2 × Low (0.6) to High (0.9) × High collective risk			-0.027^{***}
			(0.008)
Constant	0.903^{***}	0.884^{***}	0.906^{***}
	(0.001)	(0.002)	(0.003)
Observations	2644	2644	2644

Note: SEs in parentheses. * $p < 0.05$, ** $p < 0.01$, *** $p < 0.001$

2.5 H6: Effects of normative and empirical expectations

2.5.1 Analytical strategy

To understand why cooperation changes according to expectations, in [9] k-means clustering was used to generate a stringent classification of subjects based on their responsiveness to empirical and normative expectations (see Supplementary Information Section 1.4). In Wave 2 we replicate these analyses to test whether we can extract the same behavioral types and test the hypotheses that (H6a) increasing normative expectations increases contributions and (H6b) increasing empirical expectations increases contributions for some and decreases contributions for others.

2.5.2 Results

The original analyses with data from Wave 1 revealed five behavioral types: empirical cooperators (11.6%) who increase their contributions when they think others do; normative cooperators (14.1%) who increase their contributions when they think others believe they ought to cooperate; social norm followers (10.9%) who are influenced both by empirical and normative expectations; unconditional participants (37%) who do not change their behavior in response to expectations; and threshold-driven participants (26.5%), who decrease their contributions when others contribute > 50 and increase their contributions when others contribute < 50 (i.e., they are negatively influenced by empirical expectations).

The distribution of contributions for the different scenarios is similar to Wave 1 and the the k-means clustering attracts the same behavioral types (compare Figure S5A and Figure 2 in [9]). In Wave 2, 9% is classified as empirical cooperator, 12% as normative cooperator, 14% as social norm follower, 32% as unconditional participant, and 33% as threshold-driven.

Increasing normative expectations yields an 18 points average increase in the conditional contribution when empirical expectations are low ($se = 0.491$, $p < 0.001$) and an 11 point increase when empirical expectations are high ($se = 0.491$, $p < 0.001$). Depending on the

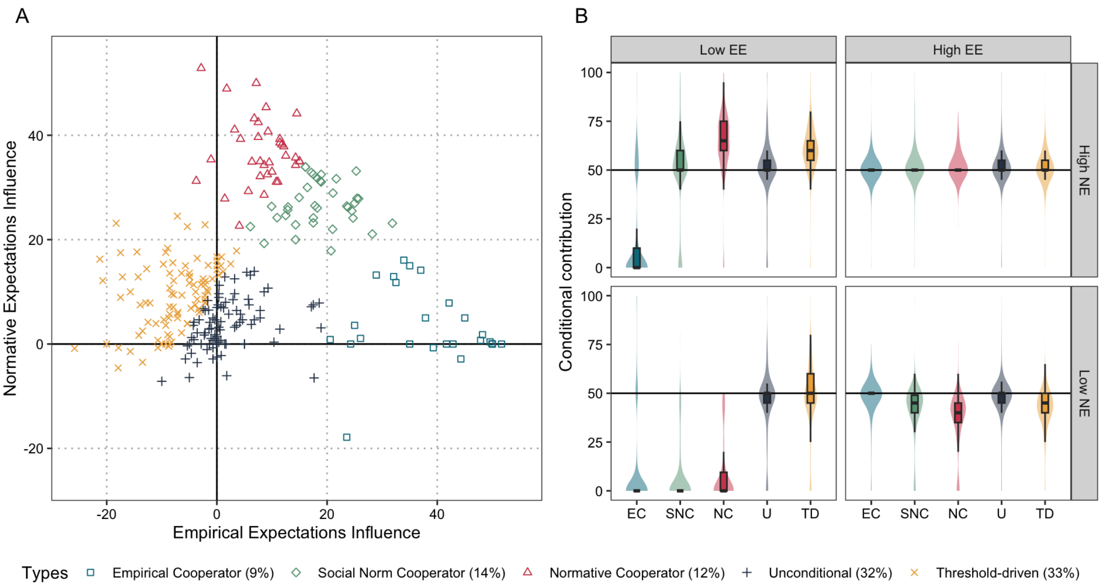

Figure S5: Conditional contributions and behavioral types in Wave 2

behavioral type conditional contributions increase by 2–52 points, but for each type the effect is positive. This is in line with the general positive effect predicted by hypothesis H6a.

More specifically, when empirical expectations are low, the increase in conditional contributions is mainly driven by social norm cooperators (who increase their contributions from 6 points to 48 points, $se = 0.970$, $p < 0.001$) and normative cooperators (who increase their contributions from 8 points to 64 points, $se = 1.024$, $p < 0.001$). Empirical cooperators slightly increase their contributions, but still contribute little overall (from 9 points to 14 points, $se = 1.192$, $p < 0.001$). Unconditional types increase their cooperation from 45 to 50 points ($se = 0.646$, $p < 0.001$) and Threshold-driven types from 48 to 60 points ($se = 11.445$, $p < 0.001$). Normative cooperators and threshold-driven types thus increase their contributions significantly beyond 50, compensating for the people contributing less than 50.

When empirical expectations are high, Normative cooperators increase their contributions the most (from 37 to 52 $se = 1.024$, $p < 0.001$), followed by social norm followers (from 41 to 51, $se = 0.970$, $p < 0.001$) and threshold-driven types (from 42 to 51, $se = 0.630$, $p < 0.001$). The Unconditional types and the Empirical cooperators both increased their contributions by 3 points from 48 to 51 ($se = 0.646$, $p < 0.001$ and $se = 1.192$, $p = 0.046$, respectively).

Increasing empirical expectations does not lead to overall increases in conditional contributions. Particularly, increasing empirical expectations leads to an overall increase of 11 points in the conditional contribution when normative expectations are low ($se = 0.491$, $p < 0.001$, but does not change the average conditional contributions when normative expectations are high. When normative expectations are high, average conditional contributions are 51 points regardless of the empirical expectations ($b = 0.104$, $se = 0.491$ $p = 0.833$).

When normative expectations are low, increasing empirical expectations leads to large increases in the contributions of the empirical cooperators (from 9 to 48, $se = 1.192$, $p < 0.001$), social norm followers (from 6 to 41, $se = 0.970$, $p < 0.001$), and normative cooperators (from 8 to 37, $se = 1.024$, $p < 0.001$). The contributions of the Unconditional types slightly increase from 45 to 48 ($se = 0.646$, $p < 0.001$). The Threshold-driven types, however, decrease their contribution by 6 points from 48 to 42 ($se = 0.630$, $p < 0.001$).

When normative expectations are high, increasing the empirical expectations leads to a large increase only for the empirical cooperators, who increase their contributions from 14 to 51 ($se = 1.192$, $p < 0.001$). Social norm followers (from 48 to 51, $se = 0.970$, $p = 0.001$) and unconditional types (from 50 to 52, $se = 0.646$, $p = 0.013$) slightly increase their contributions. On the other hand, the normative cooperators decrease their contributions by 12 points from 64 to 52 ($se = 1.024$, $p < 0.001$) and the threshold-driven types decrease their contributions by 9 points from 60 to 51 ($se = 0.630$, $p < 0.001$).

Hence, as Hypothesis H6b predicted, increasing empirical expectations increases the contributions for some, but decreases them for others. However, it should be noted that when contributions decrease under high normative expectations, this is because the normative cooperators and threshold-driven types initially overcompensated for the non-contributors to reach the threshold. Their decrease does not result in contributions below 50, so the overall group behavior remains cooperative for all behavioral types. The threshold-driven types only decrease their contributions to levels below 50 when normative expectations are low.

Table S10: Conditional contributions per behavioral type in Wave 2

	Model 1	Model 2
High EE and High NE[1]	17.683***	5.868***
	(0.471)	(0.617)
High EE and Low NE[1]	10.326***	2.480***
	(0.471)	(0.617)
Low EE and High NE[1]	17.618***	4.390***
	(0.471)	(0.617)
Empirical[2]		−32.754***
		(1.913)
Social norm[2]		−35.610***
		(1.645)
Normative[2]		−34.123***
		(1.710)
Threshold-driven[2]		4.345**
		(1.258)
High EE and High NE × Empirical		34.194***
		(1.320)
High EE and High NE × Social norm		38.180***
		(1.135)
High EE and High NE × Normative		37.725***
		(1.180)
High EE and High NE × Threshold-driven		−3.114***
		(0.868)
High EE and Low NE × Empirical		35.270***
		(1.320)
High EE and Low NE × Social norm		31.757***
		(1.135)
High EE and Low NE × Normative		25.756***
		(1.180)
High EE and Low NE × Threshold-driven		−8.394***
		(0.868)
Low EE and High NE × Empirical		−0.135
		(1.320)
Low EE and High NE × Social norm		36.508***
		(1.135)
Low EE and High NE × Normative		50.596***
		(1.180)
Low EE and High NE × Threshold-driven		6.513***
		(0.686)
Constant	31.252***	41.745***
	(0.644)	(0.894)
Random intercept subjects	77.376***	5.121***
	(7.965)	(2.659)
Random intercept round number	79.732	4.565***
	(4.654)	(2.269)
Residual variance	255.715***	2.409***
	(4.350)	(2.503)
Observations	9216	9216

Note: SEs in parentheses. [1] The reference treatment is Low EE and Low NE; [2] The reference treatment is Unconditional cooperator. * $p < 0.05$, ** $p < 0.01$, *** $p < 0.001$

3 Replication of original hypotheses

Table S11: Summary of results of original (W1) and replication study (W2).

Original hypotheses		W1	W2
1a	Contributions are positively associated with empirical expectations (EE)	+	+
1b	Contributions are positively associated with normative expectations (NE)	+	0
2	Contributions increase when manipulated EE & NE are higher	+	+
3a	Subjects are more likely to punish uncooperative subjects	+	+
3b	Subjects expect others to punish uncooperative subjects more	+	+
4	Behavioral change is faster in Low to High treatment	+	−

While we expected Covid-19 to result to overall increases, the original correlations and causal relations between risk, norms, and behavior should replicate. Wave 2 replicates most of the results of Wave 1, but there are some important differences.

- The positive association between normative expectations and contributions found in Wave 1 could not be replicated in Wave 2. This effect disappeared after controlling for personal normative beliefs.
- Like in Wave 1, increases in empirical and normative expectations increase individual contributions also in Wave 2 (confirming the original hypothesis). It should be noted, however, that in Wave 2 normative expectations alone are responsible for the increased contributions. As long as normative expectations are high, it does not matter whether empirical expectations are high or low–average contributions are above 50 in both scenarios.
- Wave 1 found that behavioral change occurs fast in Low High, but that norms provide an inertia effect on behavior in High Low. This effect could not be replicated in Wave 2. The contribution in High Low drops immediately and significantly when risk changes, to levels even below the baseline of Low High.

Detailed results per hypothesis are presented in the next sections.

3.1 H1: EE and NE as predictors of contribution

To test if Wave 2 replicates the results of Wave 1 (see Table 1 and Table S3 in [9]) we tested the hypothesis that contributions are positively correlated with both empirical expectations (H1a) and normative expectations (H1b). Just like in Wave 1, empirical expectations predict contributions (see Table S12). Other than in Wave 1, however, the effect of normative expectations is no longer significant after controlling for personal normative beliefs (Models 2–4).

Table S12: Predictors of contribution (W2).

	Model 1	Model 2	Model 3	Model 4
Empirical expectations	0.717***	0.586***	0.592***	0.548***
	(0.075)	(0.079)	(0.080)	(0.076)
Normative expectations	0.364***	0.093	0.078	0.077
	(0.074)	(0.071)	(0.071)	(0.069)
Personal normative beliefs		0.545***	0.534***	0.525***
		(0.067)	(0.065)	(0.061)
Social Value Orientation angle			0.052	0.052
			(0.031)	(0.032)
Risk preferences			−0.589**	−0.552**
			(0.202)	(0.202)
Autism Spectrum Quotient			−0.049	−0.043
			(0.054)	(0.057)
Big Five:				
Extraversion			−0.077	−0.081
			(0.050)	(0.052)
Agreeableness			−0.011	−0.008
			(0.077)	(0.077)
Conscientiousness			0.026	0.007
			(0.069)	(0.073)
Neuroticism			−0.013	−0.043
			(0.047)	(0.054)
Openness			0.120*	0.128*
			(0.054)	(0.055)
Low (0.6) to High (0.9)				1.070
				(0.929)
High collective risk				2.625***
				(0.650)
Low (0.6) to High (0.9) × High coll. risk				−0.924
				(0.815)
Age				−0.039
				(0.062)
Gender				
Female				0.977
				(0.752)
Other				−1.291
				(1.118)
Student				−1.280
				(1.203)
Experienced				−0.105
				(0.154)
Political orientation (1-7)				0.167
				(0.190)
Constant	−5.309	−13.052***	−13.859**	−10.842
	(3.635)	(3.341)	(5.319)	(5.723)
Observations	7748	7748	7748	7748

Note: SEs in parentheses. SEs adjusted for 293 clusters according to individual. The reference category in Model 4 is High-Low low collective risk. * $p < 0.05$, ** $p < 0.01$, *** $p < 0.001$

3.2 H2: Contribution according to manipulated expectations

To test if Wave 2 replicates the results of Wave 1 (see Table S4 in [9]) we tested the original hypothesis that contributions increase if we manipulate the empirical and normative expectations to be higher. As in Wave 1, increasing either empirical or normative expectations increases contributions, supporting hypothesis 2 (see Table S13). The mean contribution is 32.85, 95% CI [30.41, 35.29] in response to low empirical and low normative expectations, increases to 43.69, 95% CI [42.74, 44.64] under high empirical and low normative expectations, and increases further to 51.32, 95% CI [49.47, 53.18] under low empirical and high normative expectations or 51.43, 95% CI [50.79, 52.07] when both empirical and normative expectations are high. There is one notable difference compared to Wave 1, namely that normative expectations are entirely responsible for the change in contributions. As long as normative expectations are high, contributions are the same regardless of whether empirical expectations are high ($b_{dif} = 0.104$, $se = 0.903$, $p = 0.909$).

Table S13: Contribution according to manipulated expectations (W2).

	DV: Cond. contribution
High EE and High NE	18.576***
	(1.252)
High EE and Low NE	10.842***
	(1.193)
Low EE and High NE	18.472***
	(1.266)
Constant	32.851***
	(1.241)
Observations	8788

Note: SEs in parentheses. SEs adjusted for 293 clusters according to individual. The reference treatment is Low EE and Low NE. * $p < 0.05$, ** $p < 0.01$, *** $p < 0.001$

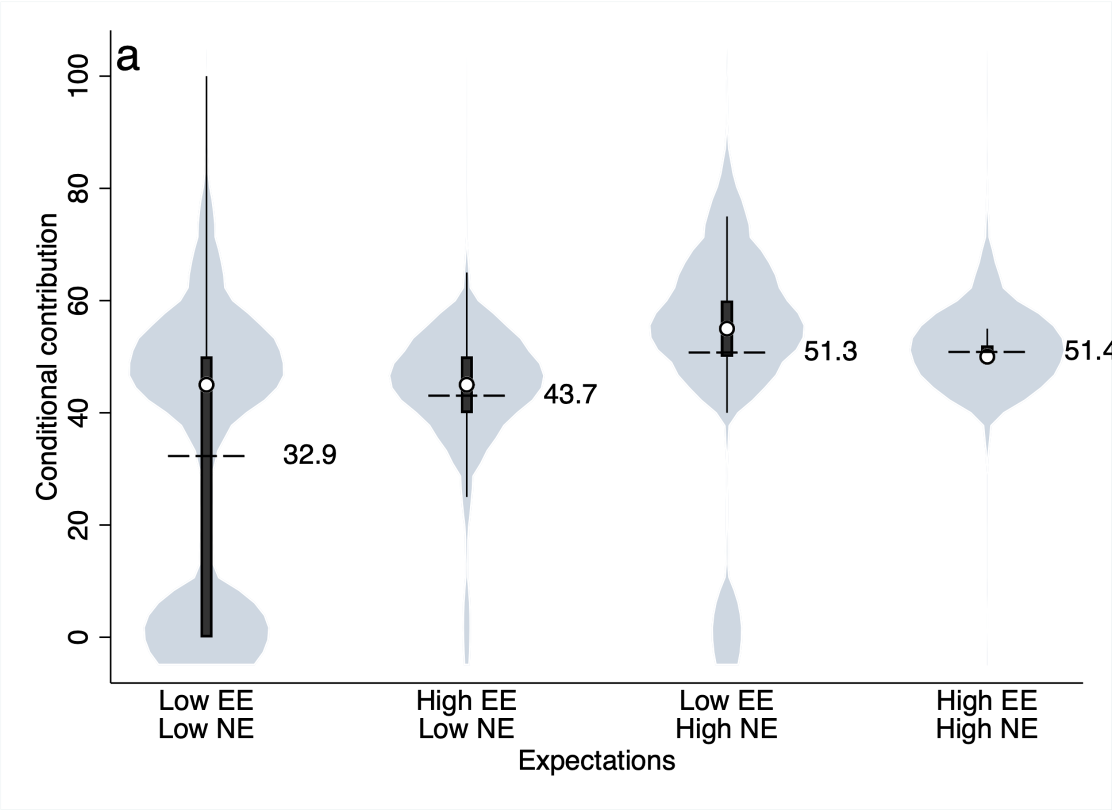

Figure S6: Violin plot of distribution of conditional contributions according to EE and NE
Note: High expectations means that the majority contributes $\geq$ 50 points; low expectations means that the majority contributes $<$ 50 points.

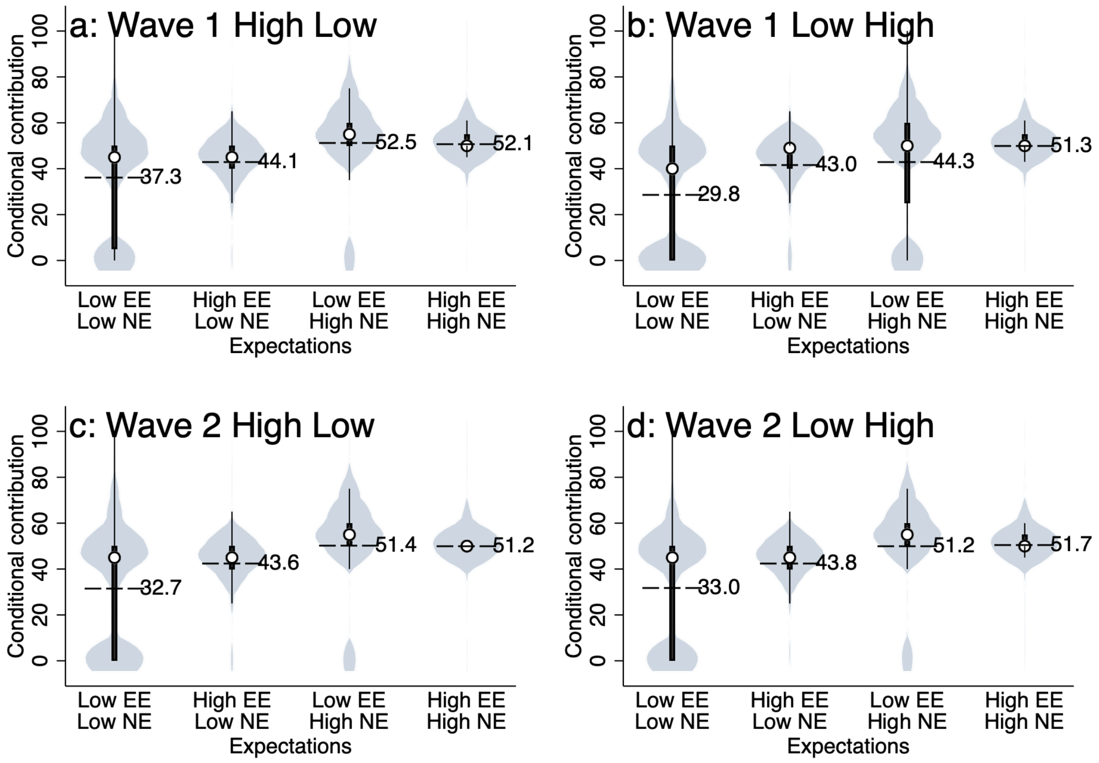

Figure S7: Violin plot of conditional contributions according to EE and NE by Wave and Treatment

Note: High expectations implies at least 50 points and low expectations implies less than 50 points. $N = 575$ individuals repeatedly measured during the experiment

3.3 H3: Punishment behavior and beliefs

The third hypothesis relates to punishment (A3a) and expectations of punishment (A3b). Both results are replicated in Wave 2 (compare Table S5 and S7 in [9] to Tables S14 and S15). Low contributors (< 50), irrespective of risk, are punished with a higher intensity (5.8, 95% CI [5.34, 6.26]) than those who contribute 50 (2.27, 95% CI [1.90, 2.64]) or more (2.15, 95% CI [1.75, 2.54] points (Hypothesis 3a) and subjects expect low contributors to be punished with a higher intensity (6.58, 95% CI [6.17, 6.98]) than higher contributors (contribute 50: 3.55, 95% CI [3.13, 3.97]; contribute more than 50: 2.88, 95% CI [2.44, 3.33] (Hypothesis 3b).

Table S14: Punishing points allocated by collective risk probability and treatment

	Model 1	Model 2
Less than 50	3.655***	4.080***
	(0.308)	(0.445)
50	0.127	0.007
	(0.145)	(0.218)
Low (0.6) to High (0.9)		0.203
		(0.405)
Less than 50 × Low (0.6) to High (0.9)		−0.848
		(0.615)
50 × Low (0.6) to High (0.9)		0.239
		(0.290)
Constant	2.145***	2.044***
	(0.202)	(0.291)
Observations	825	825

Note: SEs in parentheses. SEs adjusted for 275 clusters according to individual. * $p < 0.05$, ** $p < 0.01$, *** $p < 0.001$

Table S15: Beliefs about punishing points by collective risk probability and treatment

	Model 1	Model 2
Less than 50	3.695***	3.715***
	(0.297)	(0.414)
50	0.669***	0.504*
	(0.163)	(0.256)
Low (0.6) to High (0.9)		−0.057
		(0.453)
Less than 50 × Low (0.6) to High (0.9)		−0.041
		(0.596)
50 × Low (0.6) to High (0.9)		0.330
		(0.326)
Constant	2.884***	2.912***
	(0.226)	(0.328)
Observations	825	825

Note: SEs in parentheses. SEs adjusted for 275 clusters according to individual. * $p < 0.05$, ** $p < 0.01$, *** $p < 0.001$

3.4 H4: Faster behavioral change in Low to High Treatment

Table S16: Comparison test of cooperation level for round 15 among treatments

	DV: Relative contribution
High (0.9) to Low (0.6)	-6.683^{***}
	(1.697)
Constant	1.850
	(1.183)
Observations	276

Note: SEs in parentheses. Contribution in round 15 relative to baseline in round 14. * $p < 0.05$, ** $p < 0.01$, *** $p < 0.001$

Table S17: Dynamics of cooperation after the change in risk by treatment (round 15-28)

	DV: Relative contribution
Low (0.6) to High (0.9)	4.975^{***}
	(1.452)
Round recode	-0.212
	(0.134)
Low (0.6) to High (0.9) × Round recode	0.139
	(0.140)
Constant	-3.914^{**}
	(1.305)
Observations	3805

Note: SEs in parentheses. SEs adjusted for 283 clusters according to individual. Round hsa been recoded so that Round 15 is 0 and Round 28 is 14. * $p < 0.05$, ** $p < 0.01$, *** $p < 0.001$

The final hypothesis in [9] predicted that behavioral change would occur faster in the Low High Treatment than in the High Low Treatment. We follow the analytical strategy used in [9] that used the round 14 contributions in the two treatments to provide baseline contribution levels under low and high risk. These baselines provide a reference level that, if people only respond to the change in incentives due to the change in risk, contributions should achieve in round 15. That is, round 14 contributions in High Low and round 15 contributions in Low High (both under high risk) should be similar and *vice versa.* The analysis tests in which treatment—High Low or Low High—round 15 contribution gets closer to the baseline contribution level.

In the original study, the contributions in round 15 of Low High were essentially the same as contributions in round 14 of High Low. The contribution levels of High Low, instead, did not collapse to the contribution levels in round 14 of Low High but remained significantly larger (see Table S12 in [9]). In Wave 2, we replicate the finding that the contributions in round 15 of Low High were essentially the same as contributions in round 14 of High Low (52.72 vs 50.87) ($b = 1.850$, $p = 0.119$, 95% CIs $[-.48, 4.18]$) (Table S16). However, the contribution levels of High Low did not just collapse to the contribution levels of Low High in round 14, but became even lower (45.21 vs 50.04) ($b = -4.833$, $p < 0.001$, 95% CIs $[-7.23, -2.44]$). Hence, in Wave 1 contributions in High Low did not follow the absolute risk level only, but remained higher after experience with high risk. In Wave 2, the contributions in High Low cannot be explained from the risk level alone either, but this time subjects strongly responded to the news that risk decreased.

Subsequently, the rate of change from rounds 15-28 was compared across the two treatments.

The original study found a decrease for High Low, while contributions remain stable for Low High (see Table S13 in [9]). In Wave 2, we find no significant change in contributions in High Low ($b = -0.212$, $p = 0.113$, 95% CIs $[-0.47, 0.05]$, nor for Low High ($b = -0.073$, $p = 0.091$, 95% CIs $[-0.16, 0.01]$ (Table S17).

Combined, this indicates that like in the original study contributions in Low High immediately reach the baseline level and remain stable afterwards, while in High Low the contributions immediately jump to a level below the Low High baseline and remain stable after that. In other words, in Wave 2 the behavioral change is actually faster for the High Low treatment than for the Low High treatment, and the difference is more severe. Hypothesis 4 is not supported, but instead we observe the opposite effect.

4 Additional summary statistics and analyses

4.1 Summary statistics

Table S18: Summary statistics by treatment and overall, round 1 and 28

Variable	Round 1			Round 28		
	High Low	Low High	Overall	High Low	Low High	Overall
Number of subjects	146	147	293	137	138	275
Age (years)	24.45	24.49	24.47	24.65	24.47	24.56
	(8.24)	(9.01)	(8.62)	(8.45)	(9.00)	(8.72)
Female (prop.)	0.55	0.48	0.52	0.57	0.48	0.52
	(0.51)	(0.51)	(0.51)	(0.51)	(0.52)	(0.51)
Student (prop.)	0.84	0.82	0.83	0.83	0.83	0.83
	(0.37)	(0.38)	(0.38)	(0.38)	(0.38)	(0.38)
Experienced[a] (prop.)	0.76	0.69	0.73	0.77	0.69	0.73
	(0.43)	(0.46)	(0.45)	(0.42)	(0.46)	(0.44)
Political orientation[b] (1-7)	3.26	3.33	3.29	3.26	3.33	3.29
	(1.53)	(1.54)	(1.53)	(1.55)	(1.57)	(1.56)
SVO angle[c]	25.12	27.22	26.17	25.82	26.92	25.88
	(11.63)	(11.55)	(11.62)	(11.67)	(11.64)	(11.69)
Risk preferences[d]	2.32	2.26	2.29	2.31	2.25	2.28
	(1.53)	(1.58)	(1.55)	(1.51)	(1.57)	(1.54)
ASQ[e]	17.59	19.06	18.33	17.47	18.88	18.17
	(5.68)	(6.00)	(5.88)	(5.73)	(5.85)	(5.82)
Big Five						
Extraversion	26.33	26.68	26.51	26.30	26.86	26.58
	(6.61)	(6.56)	(6.57)	(6.68)	(6.54)	(6.61)
Agreeableness	33.90	33.76	33.83	33.85	33.75	33.80
	(4.33)	(4.55)	(4.44)	(4.42)	(4.62)	(4.52)
Conscientiousness	32.99	33.35	33.17	33.16	33.54	33.35
	(6.21)	(5.30)	(5.77)	(6.31)	(5.21)	(5.78)
Neuroticism	22.00	22.42	22.21	21.80	22.45	22.12
	(6.62)	(6.93)	(6.77)	(6.59)	(6.91)	(6.75)
Openness	38.32	38.10	38.21	38.14	38.04	38.10
	(5.90)	(5.93)	(5.91)	(5.87)	(5.95)	(5.90)

Note: Parentheses show standard deviations. [a] Experience with experiments; [b] higher score indicates more right-wing; [c] Social Value Orientation; [d] higher value indicates more risk-seeking; [e] Autism Spectrum Quotient.

To check whether there are differences between our samples in the High Low and the Low High treatments, we conducted a series of 13 unpaired t-tests comparing the samples at round 1 in the two treatments. The only treatment difference pertains to the score on the Autism Spectrum Quotient (17.59 vs 19.06, $m_d = -1.47$, $t(291) = -2.158$, $p = 0.032$). The other comparisons range between p-values of [0.121, 0.970] ($m = 0.594$, $sd = 0.267$).

To check whether there are differences between the samples of Wave 1 and Wave 2, we conducted another series of 13 unpaired t-tests comparing the samples of the two waves at round 1. The samples differ significantly for age (30.09 vs 24.47, $m_d = 5.62$, $t(577) = 6.858$, $p < 0.001$), the proportion of students (0.50 vs 0.83, $m_d = -0.33$, $t(577) = -8.860$, $p < 0.001$), and the proportion of experienced subjects (0.43 vs 0.73, $m_d = -0.30$, $t(577) = -7.67$, $p < 0.001$). The

other 10 comparisons have p-values ranging between [0.156, 0.756] ($m = 0.397$, $sd = 0.257$).

All analyses control for these sociodemographic and psychological indicators.

4.2 Summary statistics dropout

Table S19: Dropout by the end of the experiment

Variable	Dropout by round 28		
	High Low	Low High	Overall
Number of subjects	9	9	18
Age (years)	21.44	24.78	23.11
	(2.65)	(9.67)	(7.09)
Female (prop.)	0.33	0.56	0.44
	(0.50)	(0.53)	(0.51)
Student (prop.)	1.00	0.78	0.89
	(0.00)	(0.44)	(0.32)
Experienced[a] (prop.)	0.56	0.78	0.67
	(0.53)	(0.44)	(0.49)
Political orientation[b] (1-7)	3.33	3.22	3.28
	(1.22)	(0.97)	(1.07)
SVO angle[c]	29.53	31.85	30.69
	(10.57)	(9.06)	(9.62)
Risk preferences[d]	2.56	2.44	2.50
	(1.88)	(1.67)	(1.72)
ASQ[e]	19.44	21.89	20.67
	(4.69)	(7.83)	(6.39)
Big Five			
Extraversion	26.78	24.00	25.39
	(5.61)	(6.56)	(6.09)
Agreeableness	34.56	33.89	34.22
	(2.79)	(3.52)	(3.10)
Conscientiousness	30.44	30.56	30.50
	(3.84)	(6.23)	(5.02)
Neuroticism	25.11	21.89	23.50
	(5.64)	(7.72)	(7.18)
Openness	41.11	39.00	40.06
	(6.05)	(5.87)	(5.89)

Note: Parentheses show standard deviations. [a] Experience with experiments; [b] higher score indicates more right-wing; [c] Social Value Orientation; [d] higher value indicates more risk-seeking; [e] Autism Spectrum Quotient.

To test whether dropout in our study was random or not, we compare the sample composition within each treatment at round 1 and round 28. If dropout is random and/or small enough not to substantially shift the sample composition, then no average differences should be found. We undertake this comparison on each of the 13 variables for both treatments using a series of unpaired t-tests. There is no selection in dropout. The p-values for the High Low treatment range between [0.790, 0.979] ($m = 0.859$, $sd = 0.069$). Those for the Low High treatment range between [0.779, 0.988] ($m = 0.909$, $sd = 0.077$).

4.3 Individual predictors of Empirical Expectations Influence and Normative Expectations Influence

Table S20: Individual predictors of EE Influence and NE Influence

	DV: EE influence	DV: NE influence
Personal normative beliefs	−0.046	0.037
	(0.055)	(0.057)
Social Value Orientation angle	0.020	−0.004
	(0.077)	(0.063)
Risk preferences	0.338	−0.484
	(0.522)	(0.475)
Autism Spectrum Quotient	0.190	0.128
	(0.176)	(0.164)
Big Five		
Extraversion	−0.139	−0.117
	(0.154)	(0.135)
Agreeableness	0.061	−0.055
	(0.193)	(0.184)
Conscientiousness	−0.323	0.241
	(0.166)	(0.136)
Neuroticism	−0.384*	−0.083
	(0.175)	(0.125)
Openness	0.087	0.031
	(0.172)	(0.134)
Age	−0.152	−0.305*
	(0.154)	(0.119)
Gender		
Female	0.398	−3.713*
	(2.110)	(1.598)
Other	−8.209	−0.064
	(6.493)	(15.622)
Student	−6.906*	−0.700
	(3.154)	(3.077)
Experienced	0.074	0.116
	(0.509)	(0.358)
Political orientation (1-7)	−0.557	−0.133
	(0.624)	(0.483)
Constant	31.603*	17.744
	(14.693)	(12.447)
Observations	2215	2215

Note: SEs in parentheses. SEs adjusted for 293 clusters according to individual. * $p < 0.05$, ** $p < 0.01$, *** $p < 0.001$

4.4 Dynamics in social norm strength and its components

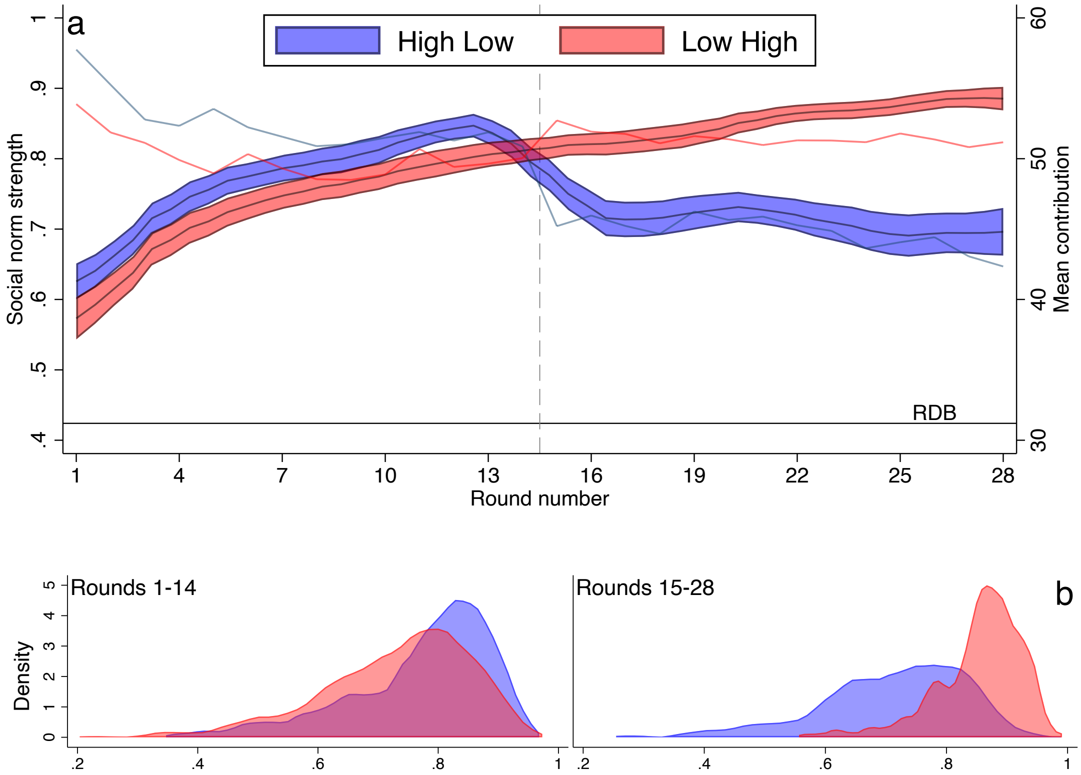

Figure S8: Social norm and contribution dynamics in Wave 2 (cf. Figure 3 in [9])

Note: [a] Norm strength (consistency × accuracy × specificity) as a function of the round. RDB indicates the Random Decision Baseline of 0.424 (mean identified using 1000 simultaions); the norm strength that would be observed if subjects made their decisions randomly for contribution, empirical expectation, normative expectations, and personal normative beliefs. Shaded areas indicate 95% CIs with one observation per group. Solid lines without shading indicate mean contributions (blue: High Low; red: Low High). [b] Distribution of norm strengths in the two stages of the experiment at the group level (one observation per group).

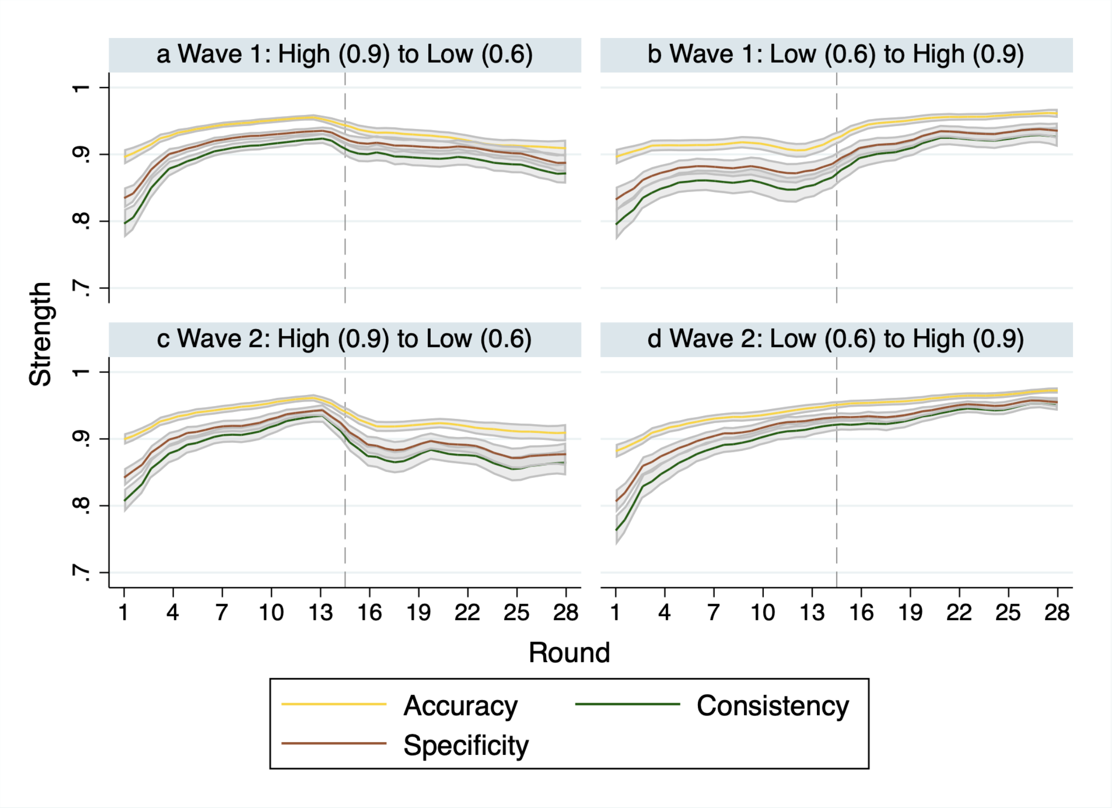

Figure S9: Social norm strength components by round and broken down by treatment
Note: [a] Wave 1: High to Low treatment; [b] Wave 1: Low to High treatment; [c] Wave 2: High to Low treatment; [d] Wave 2: Low to High treatment. Means plotted. Shaded areas indicate 95% CIs with one observation per group.

4.5 Dynamics in contributions, beliefs, and social expectations

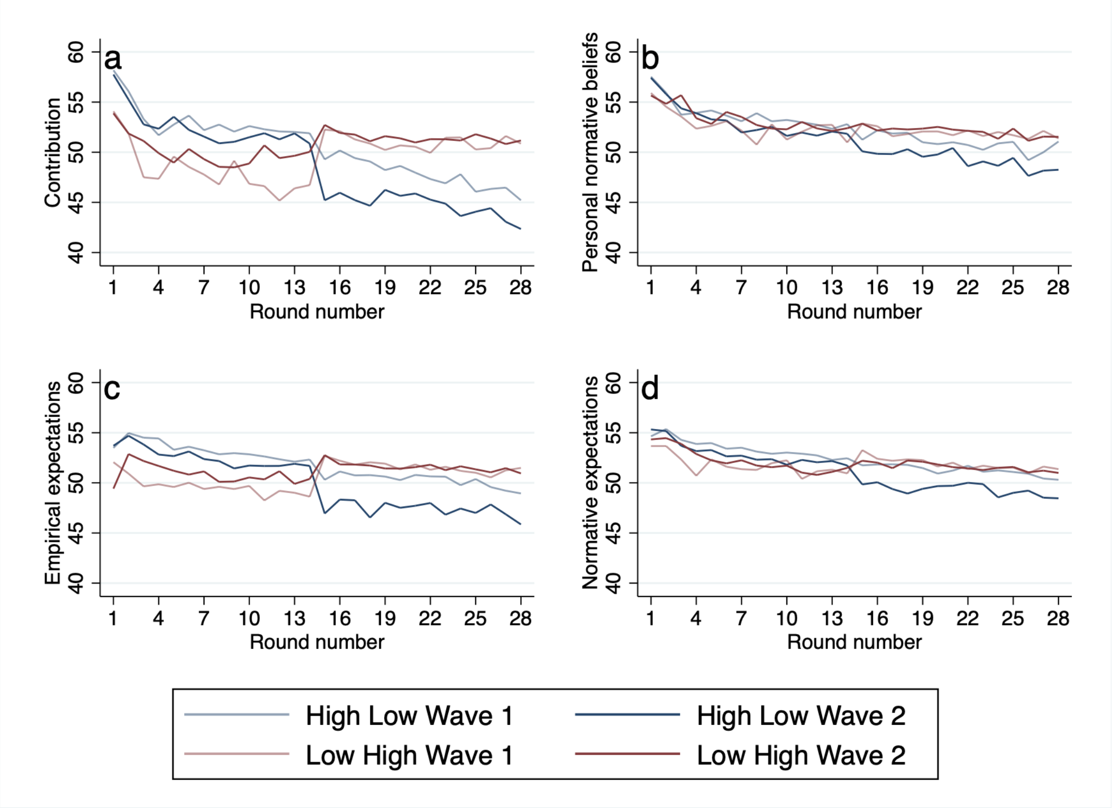

Figure S10: Contributions and expectations by round according to treatment
Note: [a] Mean contribution by round; [b] Mean personal normative beliefs by round; [c] Mean empirical expectations by round; [d] Mean normative expectations by round.

4.6 Groups reaching threshold according to social norm strength

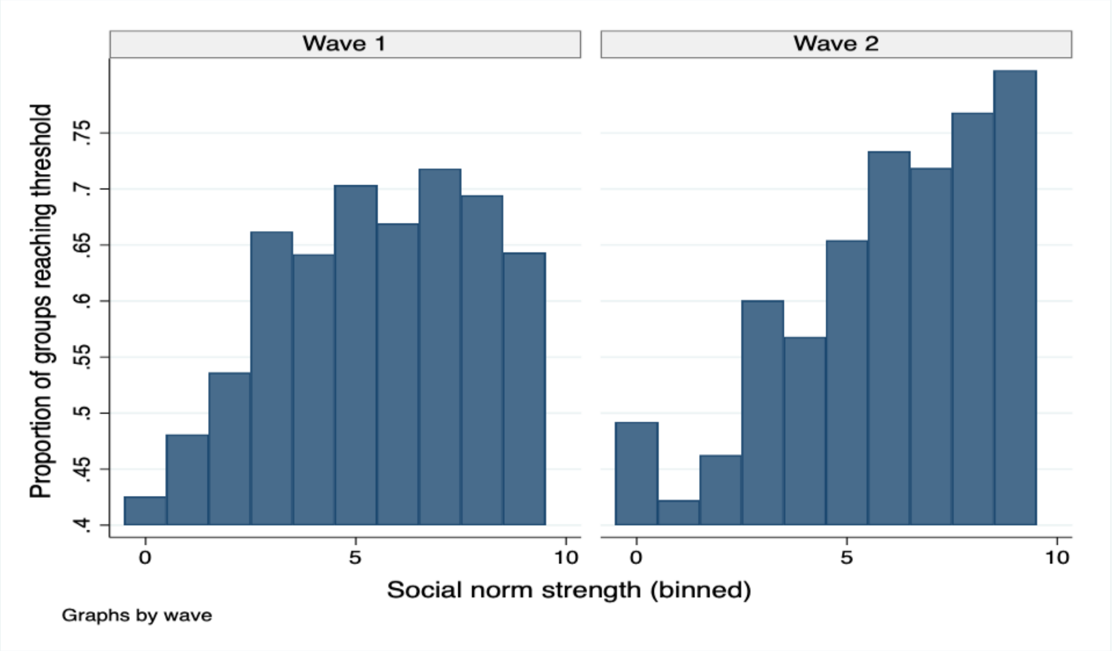

Figure S11: Proportion of groups reaching threshold by social norm strength and wave
Note: Social norm strength grouped into ten nearly equal sets of 258 or 259 group-level observations with either 258 or 259. 0 contains social norm strengths [0.228,0.593], $N_{w1} = 134$ and $N_{w2} = 124$; 1 contains [0.593,0.663], $N_{w1} = 131$ and $N_{w2} = 128$; 2 contains [0.663,0.713], $N_{w1} = 140$ and $N_{w2} = 119$; 3 contains [0.713,0.752], $N_{w1} = 139$ and $N_{w2} = 120$; 4 contains [0.752, 0.783], $N_{w1} = 134$ and $N_{w2} = 125$; 5 contains [0.783,0.813], $N_{w1} = 128$ and $N_{w2} = 130$; 6 contains [0.813, 0.837], $N_{w1} = 139$ and $N_{w2} = 120$; 7 contains [0.837, 0.862], $N_{w1} = 117$ and $N_{w2} = 142$; 8 contains [0.862,0.891], $N_{w1} = 121$ and $N_{w2} = 138$; 9 contains [0.891, 1], $N_{w1} = 84$ and $N_{w2} = 175$.

Table S21: Groups reaching the threshold according to norm strength

	Model 1	Model 2
Norm strength	3.628***	2.117***
	(0.346)	(0.370)
Wave 2		0.035
		(0.086)
Low (0.6) to High (0.9)		0.262**
		(0.087)
High collective risk		1.205***
		(0.091)
Constant	−2.233***	−1.780***
	(0.263)	(0.275)
Observations	2588	2588

Note: SEs in parentheses. Logistic regression used with one observation at the group-level per rounds. Groups with 3 or more inactive/excluded subjects are removed. * $p < 0.05$, ** $p < 0.01$, *** $p < 0.001$

4.7 Individual predictors of round 14 to round 15 contribution change

Table S22: Individual predictors of contribution change in Wave 2.

	High Low	Low High
Personal normative beliefs	0.826***	−0.043
	(0.158)	(0.164)
Social Value Orientation	−0.066	−0.031
	(0.125)	(0.095)
Risk preferences	−0.978	0.611
	(0.938)	(0.714)
Autism Spectrum Quotient	0.378	−0.152
	(0.301)	(0.207)
Big Five		
Extraversion	0.316	0.019
	(0.250)	(0.187)
Agreeableness	0.388	−0.006
	(0.346)	(0.251)
Conscientiousness	−0.239	0.203
	(0.258)	(0.208)
Neuroticism	−0.137	−0.090
	(0.260)	(0.178)
Openness	−0.304	0.026
	(0.247)	(0.192)
Questionnaire: Age	0.192	0.092
	(0.210)	(0.184)
Gender		
Female	1.798	0.244
	(3.212)	(2.437)
Other	3.575	7.527
	(16.413)	(13.254)
Student	−4.427	7.859
	(4.621)	(4.518)
Experienced	−0.498	0.307
	(0.785)	(0.575)
Political orientation (1-7)	−0.433	0.947
	(0.927)	(0.770)
Constant	−48.273*	−11.190
	(23.370)	(21.209)
Observations	132	139

Note: SEs in parentheses. Dependent variable: Change in contribution (Round 15 contribution – round 14 contribution). * $p < 0.05$, ** $p < 0.01$, *** $p < 0.001$

References

[1] Cristina Bicchieri. *The grammar of society: The nature and dynamics of social norms.* Cambridge: Cambridge University Press, 2006 (cit. on p. 13).

[2] Cristina Bicchieri. “Words and deeds: A focus theory of norms”. In: *Rationality, rules, and structure* (2000), pp. 153–184 (cit. on p. 13).

[3] Cristina Bicchieri and Erte Xiao. “Do the right thing: but only if others do so”. In: *Journal of Behavioral Decision Making* 22.2 (2009), pp. 191–208 (cit. on p. 13).

[4] Robert B Cialdini, Raymond R Reno, and Carl A Kallgren. “A focus theory of normative conduct: Recycling the concept of norms to reduce littering in public places.” In: *Journal of personality and social psychology* 58.6 (1990), p. 1015 (cit. on p. 13).

[5] Rachel Croson and James Konow. “Social preferences and moral biases”. In: *Journal of Economic Behavior & Organization* 69.3 (2009), pp. 201–212 (cit. on p. 13).

[6] Michele J. Gelfand et al. “Differences between tight and loose cultures: A 33-nation study”. In: *Science* 332.6033 (2011), pp. 1100–1104. ISSN: 10959203. DOI: 10.1126/science.1197754 (cit. on p. 16).

[7] James Konow. “Fair shares: Accountability and cognitive dissonance in allocation decisions”. In: *American economic review* 90.4 (2000), pp. 1072–1092 (cit. on p. 13).

[8] Richard H Price and Dennis L Bouffard. “Behavioral appropriateness and situational constraint as dimensions of social behavior.” In: *Journal of Personality and Social Psychology* 30.4 (1974), p. 579 (cit. on p. 16).

[9] Aron Szekely et al. “Evidence from a long-term experiment that collective risks change social norms and promote cooperation”. In: *Nature Communications* 12.1 (2021). ISSN: 20411723. DOI: 10.1038/s41467-021-25734-w (cit. on pp. 29, 33, 37, 39, 42–44, 49).

[10] Erte Xiao and Daniel Houser. “Avoiding the sharp tongue: Anticipated written messages promote fair economic exchange”. In: *Journal of Economic Psychology* 30.3 (2009), pp. 393–404 (cit. on p. 13).
